# Nanomedicines and stroke: Advantages in chronic inflammation treatment and neural regeneration

**DOI:** 10.4103/NRR.NRR-D-25-00120

**Published:** 2025-08-13

**Authors:** Chuhan Liu, Yuanyuan Ran, Changbin Hu, Mengjie Wang, Ning Li, Zhi Yang, Zitong Ding, Chenye Qiao, Jianing Xi, Wei Su, Lin Ye, Zongjian Liu

**Affiliations:** 1 Beijing Rehabilitation Hospital, Capital Medical University, Beijing, China; 2 Beijing Tsinghua Chang Gung Hospital, School of Clinical Medicine, Tsinghua University, Beijing, China; 3 School of Material Science and Engineering, Beijing Institute of Technology, Beijing, China

**Keywords:** blood–brain barrier, drug delivery, hemorrhagic stroke, ischemic stroke, nanomedicine, nanotechnology, neural regeneration, neuroimmunomodulation, regenerative medicine, stroke

## Abstract

Stroke can be categorized as ischemic and hemorrhagic on the basis of its origin. The pathophysiology following a stroke is complex, and is characterized by ongoing inflammation, neuronal injury, and the accumulation of reactive oxygen species in the brain, all of which reflect a dynamic process of change. This complexity hinders achievement of significant therapeutic outcomes with standard stroke treatment procedures, limiting post-stroke recovery. This review presents an innovative post-stroke therapeutic approach that utilizes nanomedicines to modify the cerebral microenvironment. It highlights the primary roles of chronic inflammation and nerve repair issues in causing prolonged impairment in stroke patients. Traditional therapies show limited effectiveness in achieving neuroprotection, immunoregulation, and neural regeneration during the subacute and chronic phases of stroke. Therefore, effective stroke management requires the use of specific therapeutic strategies tailored to the pathological characteristics of each phase. Various types of nanomedicines possess distinct physicochemical properties and can be selected on the basis of the specific therapeutic needs. Surface-modification technologies have significantly enhanced the ability of nanomedicines to penetrate the blood–brain barrier and improve their targeting capabilities in drug administration. However, the stability, biocompatibility, and long-term safety of nanomedicines require further optimization for clinical application. Nanomedicines represent a novel approach to stroke treatment through targeted delivery and multifaceted regulatory mechanisms. These medicines provide distinct advantages, particularly in addressing chronic inflammation and promoting nerve regeneration. As a result, nanomedicines are expected to significantly improve rehabilitation outcomes and quality of life for stroke patients in the future, emerging as a crucial modality for stroke treatment.

## Introduction

Stroke has recently emerged as the leading cause of death and disability globally, becoming a major chronic non-communicable disease that substantially endangers public health (Bennett et al., 2014; Boursin et al., 2018; Wang et al., 2025). Stroke can be primarily categorized as ischemic and hemorrhagic, which includes cases of intracerebral hemorrhage and subarachnoid hemorrhage (Li et al., 2025; Xu et al., 2025). In China, ischemic stroke (IS) accounts for over 80% (81.9%) of the cases of stroke, while intracerebral hemorrhage and subarachnoid hemorrhage account for 14.9% and 3.1%, respectively (Tu and Wang, 2023). IS is a medical emergency characterized by reduced blood flow to the brain, resulting in damage to brain cells (Campbell et al., 2019; Walter, 2022). Hemorrhagic stroke (HS) refers to the rupture of blood vessels within the brain or subarachnoid space, leading to the infiltration of blood into brain tissue or ventricles directly from the ruptured vessels (Montaño et al., 2021a; Unnithan et al., 2025). This results in brain edema, compression, displacement, softening, and necrosis of brain tissue. Alternatively, blood may enter the subarachnoid space, causing cerebral vasospasm, cerebral ischemia, cerebral edema, and softening of the affected brain tissue. The consequences of a stroke are substantial and multifaceted (GBD 2019 Stroke Collaborators, 2021). These consequences can lead to physical impairments such as hemiplegia, paralysis, dysphagia, and speech impairment, which significantly diminish the patients’ quality of life (Towfighi et al., 2017; El Husseini et al., 2023; Zhang et al., 2024b). From a socio-economic perspective, the direct medical costs and indirect economic losses attributed to stroke globally exceed hundreds of billions of dollars annually (Abbott et al., 2010; Krishnamurthi et al., 2020; Denny and Gattellari, 2022). Treatment of stroke involves a complex and prolonged approach. A considerable proportion of patients continue to face challenges in improving their physical function during the later phases of recovery (Walter, 2022), and the risk of recurrent stroke and other cardiovascular events remains elevated (Kolmos et al., 2021; Lin et al., 2021; Xu et al., 2022a). Stroke survivors have a ten-fold higher risk of experiencing another stroke in comparison with the general population (Abbott et al., 2010; Burch, 2014; Hankey, 2014; Stahmeyer et al., 2019). The pathophysiological mechanisms of stroke encompass vascular injury, cellular metabolic dysfunction, oxidative stress, inflammatory responses, and compromised nerve repair and regeneration (Iadecola and Anrather, 2011; Iadecola et al., 2020). Vascular injury following IS can result in impairment of the blood–brain barrier (BBB), which is an important event in the initial phases of IS. The disintegration of the BBB begins shortly after stroke and peaks within a 72-hour time frame. This condition is marked by a reduction in the expression of tight junction proteins, such as claudin-5 and occludin, leading to increased BBB permeability (Bennett et al., 2014). Our team showed that this damage facilitates the infiltration of peripheral immune cells into the brain, while also promoting the entry of inflammatory cells and the production of inflammatory mediators. This process exacerbates brain edema and leads to further brain damage (Abbott et al., 2010; Liu et al., 2016; Ran et al., 2018). During the subacute and chronic phases, inadequate repair of the BBB and dysfunction resulting from impaired mitochondria can lead to ongoing inflammation and oxidative stress, potentially worsening brain tissue damage and obstructing the recovery of neurological function (Sheth et al., 2015). Furthermore, the BBB may remain unrepaired for weeks, months, or even years following a stroke, resulting in persistent alterations to the microenvironment and inflammatory conditions (Shichita et al., 2017; Hurd et al., 2021). Ischemia can cause impaired mitochondrial function, the release of cytochrome c, activation of apoptotic pathways, and ultimately, cell death. Excessive levels of reactive oxygen species (ROS) cause cellular damage through oxidation of biological macromolecules. The activation of inflammatory cells and pattern recognition receptors contributes to the release of inflammatory chemicals, which worsens cell damage (Farhoudi et al., 2023). Moreover, diminished expression of nerve growth factors and increased autophagy activity can affect the regeneration and repair of neurons (Nixon, 2013; Sheth et al., 2015). The intricate biological processes involved in IS are interconnected and play important roles in stroke development. The primary brain injury in HS results from the space-occupying effect and mechanical damage caused by bleeding, alongside the breakdown of blood-related components, damage to the BBB, and a range of secondary injuries, including ROS production and inflammation. These factors collectively contribute to the overall influence of stroke (Almutairi et al., 2019; Alsbrook et al., 2023; Ohashi et al., 2023). The complex pathophysiological alterations lead to diminished functionality and a decline in the quality of life for patients following a stroke. Understanding the pathophysiological causes of stroke at various time intervals is essential for effective post-stroke treatment and rehabilitation. Contemporary treatments such as tissue plasminogen activator administration, thrombectomy, angioplasty, and stent implantation can stabilize patients’ vital signs during initial care. However, many patients face challenges in achieving substantial outcomes during the later phases of rehabilitation (Zheng et al., 2016; Montaño et al., 2021b; Paul and Candelario-Jalil, 2021; Ahmed et al., 2024; Kim et al., 2025). The primary challenges of the existing therapeutic modalities include a restricted treatment window, short half-lives, inadequate biodistribution specificity, and the inability of current therapeutic drugs to penetrate the BBB (O’Collins et al., 2006; Chamorro et al., 2016; Bharadwaj et al., 2018; Samal and Segura, 2021). Despite receiving timely treatment, nearly 50% of patients are unable to live independently (Sheth et al., 2015). Importantly, throughout the entire stroke process, particularly in the subacute and chronic phases, conventional therapeutic interventions demonstrate limited efficacy in neuroprotection, immunomodulation, and synaptic plasticity (Grefkes and Fink, 2020; Yang et al., 2021b).

Since its inception in 1959, nanomedicine has emerged as a new domain of research (Kim et al., 2010; Lammers et al., 2011; Ouyang et al., 2022). Nanomedicine encompasses the design, production, and application of materials or devices utilizing nanotechnology within the range of 1 to 1000 nm for purposes such as disease prevention, diagnosis, monitoring, treatment, and repair. In the context of stroke, nanomedicine can address the challenges associated with conventional pharmaceuticals, including inadequate solubility, short half-life, and difficulties in penetrating the BBB. By using nanoscale carriers or structures, nanomedicine can improve the biodistribution, targeting, release kinetics, and bioavailability of medications (Jain, 2006; Tam et al., 2014; Dilnawaz et al., 2018; Patra et al., 2018; Selim et al., 2019; Wang et al., 2021; Farhoudi et al., 2023; Giorgi et al., 2024). Nanomedicine presents promising opportunities for the diagnosis and treatment of various diseases, and researchers are actively exploring its application for stroke treatment. This review analyzes the use of nanomedicines in stroke management, highlighting a range of nanomedicines, including liposomes, polymers, metal nanoparticles, and self-assembled peptide-based hydrophilic gels. These innovations will facilitate hemostasis, reperfusion, neuroprotection, prevention of complications, and nerve regeneration, offering novel approaches to stroke treatment. A summary of several nanomedicine experiments is provided in **Additional Table 1**.

**Additional Table 1:** Application of related nanomedicines in stroke management

Nanomedicine	Drug	Nano-carrier	Nanomedicine surface-modified substances	Animal model	Administration method/ timing	Mode of action	Reference
Metal nanomedicine	Gastrodin	Au-G5.NHAc-PS	1,3-Propane sultone (1,3-PS) is modified by a surface amino reaction	Male Sprague-Dawley rats are a model for local cerebral ischemia.	Tail vein injection/ administered three times within 7 d after surgery	It reduces the expression of certain proteins in astrocytes and hypothalamic neurons, while also improving cell damage and the arrangement of nerve fibers.	Huang et al., 2022
Metal nanomedicine	MnCO_3_, ICG	BSA nanoparticles		Male mice aged 8 wk were used to simulate an acute ischemic stroke.	Magnetic resonance and photoacoustic imaging at 0.5, 1.5, 3, and 5 h after tail vein injection/injection	The pH sensitivity of MnCO3@BSA-ICG breaks down ischemic infarct tissue, leading to the release of Mn^2+^, which binds to BSA and enhances T1 magnetic resonance imaging longitudinal relaxation. This nanomedicine enables rapid and accurate diagnosis of acute ischemic stroke. Additionally, it can assist in therapeutic decision-making and patient recovery assessments	Song et al., 2022
Organic polymer nanomedicine	NR2B9C	SHp-RBC-NP	Red blood cell membrane attached to reactive oxygen species-responsive nanoparticles; stroke homing peptide targets nanomedicine to ischemic brain tissue	Rats and mice were used to create a model of MCAO.	Tail vein injection/ administration 1, 2, 6 h after MCAO model establishment	NR2B9C, a neuroprotectant, prevents excessive nitric oxide generation by blocking PSD-95-N-methyl-D-aspartate receptor interactions. Drug circulation is prolonged by red blood cell membrane. Also, nanoparticles can be disseminated to ischemic areas.	Lv et al., 2018
Organic polymer nanomedicine	Glyburide	PLGA nanoparticles	Nerve stem cell membrane overexpressing CXCR4	C57BL/6 mice were used to model MCAO.	Injection/administration into tail vein 0, 24, and 48 h after surgery.	Glyburide protects neurons, reduces stroke-related brain edema, and cell death. High-CXCR4 neural stem cells deliver medications to the brain better than others.	Ma et al., 2019
Organic polymer nanomedicine	Cy5.5 NIRF dye, OA-Fe_3_O_4_ NPs	BCN-NPs	Biological orthogonal reagent modified BCN-NPs	A photothrombotic stroke model was created in 10-wk-old, 20-25 g male nude Balb/c mice.	A stroke mouse model received nanoparticles in its brain using a specific procedure. The infusion was 5 μL at 250 nL/min.	BCN-dual-NPs can label hMSCs and track stem cells *in vivo* using NIRF/MR dual-modal imaging technology.	Lim et al., 2019
Organic polymer nanomedicine	RES	PVP-PCL NPs		The tMCAO model was used in adult male Sprague-Dawley rats (250-300 g).	Injection of 5 mg/kg RES into the carotid artery at the start of reperfusion.	RES protects cells from blood flow restriction as an antioxidant. It removes toxic chemicals to safeguard cells.	Lu et al., 2020
Organic polymer nanomedicine	Gallic acid	O-CMC	Sodium tripolyphosphate cross-linking	Rats were used to create a model of MCAO.	Take by mouth once a day for 7 d before surgery. Dose was 50 mg/kg.	Gallic acid reduces inflammation and increases superoxide dismutase and GSH-Px, which strengthen antioxidant defenses. In ischemic brain injury, decreasing inflammation and oxidation lowers neuronal death and dysfunction.	Zhao et al., 2020
Organic polymer nanomedicine	Baicalein	PEG-PLGA NPs	RVG29 peptide	Rats (280-320 g) were used to create a model of MCAO.	Intranasal or intravenous injection on the third day after the model was set up.	Baicalein protects the brain by fighting free radicals and reducing inflammation. Meanwhile, nanoparticles help the drug reach the brain.	Li et al., 2022a
Organic polymer nanomedicine	Tetramethylpyrazine	PLGA and PEG	Peptide CFLFLF (cinnamyl-F-(D)L-F-(D)LF)	Adult male mice weighing 20-25 g and aged 6-8 wk old were used to establish the MCAO model.	Injection in tail vein: 0 h, 1 d, 4 d	The peptide CFLFLF targets neutrophils to transport the medication to the ischemic brain. Tetramethylpyrazine reduces inflammation and protects the brain.	Mu et al., 2023
Organic polymer nanomedicine	Glyburide	Poly(2,2'-thiodiethylene 3,3'-thiodipropionate) polymer	AMD3100, PEG		Tail vein injection/0, 24 and 48 h after surgery	AMD3100 surface modification targets ischemic brain tissue. Thioether groups in the poly(2,2'-thiodiethylene 3,3' thiodipropionate) polymer react with reactive oxygen species in ischemic brains, providing antioxidative effects. Additionally, glibenclamide reduces brain swelling.	Wu et al., 2022
Liposomal nanomedicine		A liposomal drug composed of phosphatidylcholine, cholesterol and monosialogangliosides		C57BL/6J mice were used to estabish a MCAO model.	Abdominal cavity injection, intravenous injection/ abdominal cavity injection 1 h before treatment, intravenous injection during surgery.	Nano-liposomes activate the Nrf2 signaling pathway and increase cell production of antioxidant proteins such as heme oxygenase-1, NQO1, and superoxide dismutase to minimize oxidative stress and protect cells.	Ahmad et al., 2022
Liposomal nanomedicine	ZL006	Liposome consisting of soy lecithin, cholesterol, 1,2-distearoyl-sn-glycero-3-phosphoethanolamine-polyethylene glycol 2000 (DSPE-PEG2000) in a specific molar ratio of 80/20/8	T7 peptide, stroke-homing peptide	Adult male Sprague-Dawley rats were used to establish a model of MCAO	Tail vein injection/ immediate post-operative administration	The BBB allows T7 peptide-modified nanocarriers to enter the brain via binding to transferrin receptor on brain capillary endothelial cells. Stroke-homing peptide aids medication delivery to ischemic regions. ZL006 reduces brain cell death from blood flow deprivation.	Zhao et al., 2016a
Liposomal nanomedicine	Valsartan	Solid lipid nanoparticles	Rhodamine B	Male albino mice, 12 wk old, were used to establish models of induced permanent stroke at the middle cerebral artery.	Administer intranasally 3 d before and 3 d after stroke induction	The nasal route crosses the BBB, and solid lipid nanoparticles govern drug release. The medication valsartan is released slowly over 72 h. Valsartan may protect the brain by decreasing angiotensin II and activating AT2 receptors.	Sabry et al., 2023
Inorganic non-metallic nanomedicine	Dl-3-n-butylphthalide	Cerium oxide nanoparticles	PEG	A 20-wk-old male C57BL/6 mouse was used to create a model of stroke.	Intravenous tail vein injection/once a day for 14 d after surgery	Cerium oxide nanoparticles shield against free radicals. They enhance antioxidant and neurovascular healing of the medicines. Dl-3-n-butylphthalid increases cerebral blood flow, promotes cell growth, lowers inflammation, and prevents cell death.	Li et al., 2022c
Inorganic non-metallic nanomedicine	Edaravone	Ceria nanoparticles	Angiopep-2; PEG	Adult male Sprague-Dawley rats were used to establish model of MCAO.	Intravenous tail vein injection/drug injection within 24 h after surgery	Angiopep-2 peptide binds to the BBB LRP receptor, allowing nanocarriers to cross. Then, Ceria nanoparticles with loaded edaravone eliminate reactive oxygen species and protect the BBB and brain cells.	Bao et al., 2018
Extracellular vesicle nanomedicine	Resolvin D2 (RvD2)	Nanovesicles derived from neutrophil membranes		C57BL/6 mouse models of MCAO	Tail vein injection, 1 h after reperfusion	Nanovesicles treat neuroinflammation and brain injury by targeting inflammatory cerebrovascular cells, releasing RvD2, inhibiting neutrophil infiltration, and reducing inflammatory markers.	Dong et al., 2019
Extracellular vesicle nanomedicine	Extracellular vesicles derived from neural progenitor cells	RGD-EVReN	Reorganization of fusion protein (RGD-4C peptide fused to the C1C2 domain of the milk fat globule-EGF factor 8 protein)	C57BL/6 mouse models of MCAO	Tail vein injection, administration 12 h after reperfusion	Targeting integrin αvβ3 reduces ischemic brain injury by delivering extracellular vesicles to the lesion area, inhibiting inflammatory signaling pathways, reducing pro-inflammatory cytokines, and inhibiting microglial activation.	Tian et al., 2021
Nanozyme	PB	PB		C57BL/6 mouse models of MCAO	Tail vein injection, 1 h after ischemia-reperfusion injury	The chemical structure of PBzyme contains iron-cyanide groups. PBzyme exhibits superoxide dismutase and catalase-like activities due to these groups. The Fe^3+^ and Fe^2+^ ions in PBzyme can modulate intracellular redox equilibrium through redox reactions, which occur as a result of their changing oxidation states.	Liu et al., 2023a
Nanozyme	Two-dimensional vanadium carbide MXene-based nanozyme	Two-dimensional vanadium carbide MXene-based nanozyme	Polyvinylpyrrolidone	Adult male Sprague-Dawley rats were used to establish a model of MCAO.	Intracerebroventricular injection (4 μL, 40 μg/mL); preoperative injection	Nanozyme simulates superoxide dismutase, catalase, peroxidase, and glutathione peroxidase activities to remove reactive oxygen species such as O_2_^+^, H_2_O_2_, and hydroxyl radicals.	Hu et al., 2022

This table provides a summary of preclinical research on nanomedicines used for stroke treatment. It includes details on the type of nanomedicine, the drug being delivered, the nanocarrier, the method of surface modification, the experimental animal model, the administration method and dosage, as well as the mechanism of action of the nanomedicine. This table aims to facilitate a quick understanding of the nanomedicines utilized in stroke research for researchers. AT2: Angiotensin II type 2 receptor; BBB: blood-brain barrier; BCN: bicyclo[6.1.0] non-3-ene; BCN-NPs: bicyclo[6.1.0] non-3-ene-modified glycosaminoglycan nanoparticles; BSA: bovine serum albumin; CXCR4: C-X-C motif chemokine receptor 4; GSH-Px: glutathione peroxidase; hMSCs: human mesenchymal stem cells; ICG: Indigo carmine green; MCAO: middle cerebral artery occlusion; MnCO_3_: manganese bismuthate; NIRF: near-infrared fluorescence; NPs; nanoparticles; NQO1: NAD(P)H quinone dehydrogenase 1; OA-Fe_3_O_4_ NPs: oleic acid-modified superparamagnetic iron oxide nanoparticles; O-CMC: O-carboxymethyl chitosan; PA: photoacoustic; PB: Prussian Blue; PEG: polyethylene glycol; PLGA: poly(lactic-co-glycolic) acid; PVP-PCL NPs: polymer nanoparticles; RES: resveratrol; tMCAO: transient middle cerebral artery occlusion.

This article aims to systematically incorporate the latest scientific advancements in nanomedicine for stroke therapy. By analyzing the pathological processes and treatment requirements for stroke, and discussing nanomedicine strategies, applications, future directions, and clinical translation, this article establishes a theoretical foundation and technical roadmap for developing new solutions for stroke therapy. The objective of this study is to expedite the clinical translation of nanomedicine in stroke neurorepair and regeneration.

## Retrieval Strategy

A computer-based online search of the PubMed database was conducted to retrieve articles published from the database’s inception to February 10, 2025. A combination of the following MeSH terms was used to maximize both search specificity and sensitivity: “ischemic stroke,” “stroke,” “hemorrhagic stroke,” “intracerebral hemorrhage,” “nanomedicine,” “nanoparticle,” “blood–brain barrier,” “drug delivery,” “extracellular vesicles,” “liposome,” “nanozyme,” “carbon-based nanomaterials,” “clinical,” and “model.” The results were further screened by reading the titles and abstracts, and studies exploring the potential mechanisms or clinical strategies of nanomedicine therapy in stroke, as well as those involving nanoengineering, were included. No restrictions on language or study type were applied.

## Pathological Mechanisms and Treatment Requirements of Stroke

Stroke is an acute cerebrovascular condition that typically results from rupture or obstruction of cerebral blood vessels, which cause damage in brain tissue due to insufficient oxygen and nutrients (Maryenko, 2024; Zhou et al., 2025). Stroke is categorized into IS, which accounts for 80% of cases, and HS, which accounts for the remaining 20%. IS commonly results from thrombosis, atherosclerosis, or cardiogenic embolism, while HS may arise from factors such as hypertension, cerebral vascular malformations, and cerebral amyloid angiopathy. Conventional therapies are often hindered by the inherent instability of medications, inadequate blood circulation, suboptimal pharmacokinetics, and immune system interactions. These challenges complicate the effective delivery of drugs to the targeted lesion area, limiting the ability of these drugs to meet treatment requirements. This section outlines the pathology and requirements associated with various phases of stroke, providing insights for the design of nanomedicines.

### Pathological characteristics and treatment requirements across various phases of ischemic stroke

#### Initial phase

**Figure 1:**
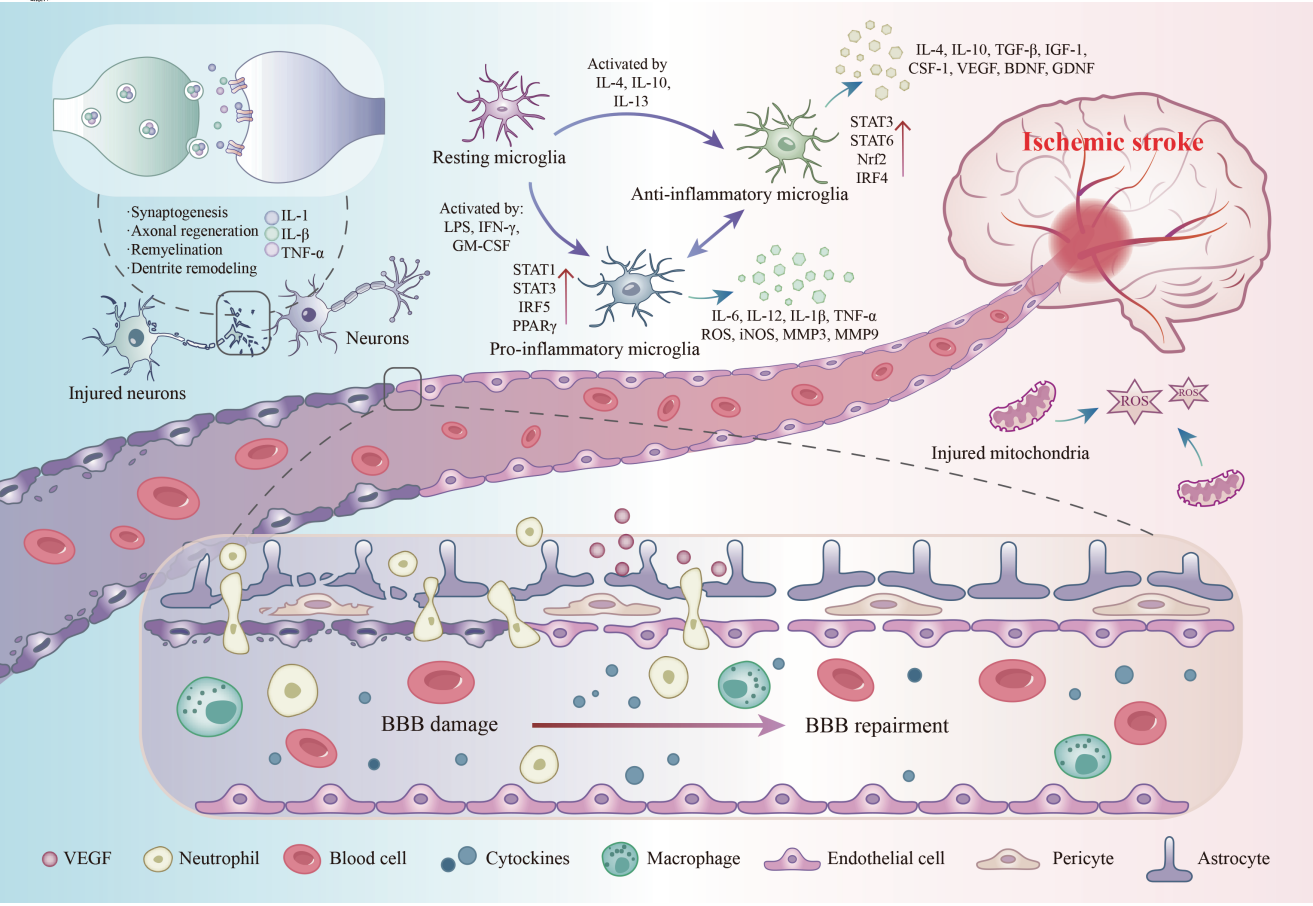
Schematic diagram of the pathophysiological mechanisms of IS: neuroinflammatory response, microglial polarization, and BBB damage and self-repair. Neuroinflammatory response: The release of inflammatory cytokines (IL-1, IL-1β, TNF-α) activates the NMDA/AMPA signaling pathways, leading to mitochondrial damage and increased levels of ROS. Microglial polarization: Stimulation by various compounds causes microglia to polarize in different directions. BBB damage and self-repair: In the early stages of IS, endothelial cell damage and the breakdown of tight junctions in the membrane result in BBB disruption, allowing peripheral inflammatory substances to enter the brain and exacerbate inflammation. In the subacute and chronic phases of IS, the release of angiogenic factors such as VEGF promotes BBB self-repair. BBB: Blood–brain barrier; BDNF: brain-derived neurotrophic factor; CSF-1: colony-stimulating factor 1; GDNF: glial cell–derived neurotrophic factor; IFN-γ: interferon-gamma; IGF-1: insulin-like growth factor-1; IL: interleukin; iNOS: inducible nitric oxide synthase; IRF: interferon regulatory factor; IS: ischemic stroke; LPS: lipopolysaccharide; MMP: matrix metalloproteinase; Nrf2: nuclear factor erythroid 2-related factor 2; ROS: reactive oxygen species; STAT: signal transducer and activator of transcription; TGF: transforming growth factor; VEGF: vascular endothelial growth factor.

Approximately 6 hours after the onset of IS, which represents the ideal time frame for pharmacological and surgical intervention, blood circulation is re-established through hemorheological reperfusion (Thiebaut et al., 2018; Powers, 2020). In the central region of ischemia, reduced perfusion leads to a significant decrease in the availability of oxygen and glucose in brain tissue, thereby diminishing the energy supply to neurons (Karaszewski et al., 2009). The deficiency of adenosine triphosphate (ATP) disrupts the normal functioning of ion pumps, resulting in an influx of Na^+^ and an outflow of K^+^, which culminates in cellular swelling, membrane damage, and, ultimately, cell death. In the ischemic penumbra, glutamate accumulates excessively in the synaptic cleft. The surplus of excitatory amino acids overactivates the N-methyl-D-aspartate (NMDA) receptors and α-amino-3-hydroxy-5-methyl-4-isoxazolepropionic acid (AMPA) receptors on neurons, leading to an influx of Ca^2+^ and the activation of several Ca^2+^-dependent enzymes. Nitric oxide synthase (NOS) and ROS-producing enzymes contribute to mitochondrial dysfunction (Thiebaut et al., 2018). Mitochondrial damage exacerbates cellular apoptosis, facilitating disruption of the BBB and invasion of inflammatory cells (**Figure 1**).

#### Acute phase

This phase lasts from 6 to 72 hours following the onset of IS (Bernhardt et al., 2017). During this period, the abrupt cessation of nutrient supply leads to reduced expression of tight junction proteins, including claudin and ZO-1, within the BBB (Abbott et al., 2010; Guruswamy and ElAli, 2017). This disruption causes an imbalance in endothelial cell signaling, particularly that involving vascular endothelial growth factor (VEGF) and other angiogenic factors. The consequent impairment of the BBB—a structure composed of endothelial cells, pericytes, and newly formed glial cells—results in loss of functionality of the BBB, increased BBB permeability (Sun and Nan, 2016), and exacerbation of cerebral edema (Candelario-Jalil, 2009; Iadecola and Anrather, 2011). In this phase, neurons, astrocytes, and microglia in the ischemic zone are activated, releasing inflammatory mediators such as tumor necrosis factor (TNF)-α, interleukin (IL)-1β, and IL-6 (**Figure 1**). Previous studies (Liu et al., 2019, 2023b; Ran et al., 2021a; Qiao et al., 2023) have indicated that these inflammatory mediators may initiate both local and systemic inflammatory responses and activate microglia, prompting their transition to a pro-inflammatory phenotype. Furthermore, ischemic injury activates Toll-like receptors (TLRs), particularly TLR4, which triggers MyD88-dependent signaling pathways, activates nuclear factor kappa-light-chain-enhancer of activated B cells (NF-κB) (Iadecola and Anrather, 2011), enhances the transcription and release of inflammatory mediators, and results in cellular damage in the brain, primarily affecting neuronal cells.

#### Subacute phase

The subacute phase of IS is defined as the interval spanning several days to weeks following the onset of the stroke (Bernhardt et al., 2017). This period is marked by intricate pathophysiological alterations in brain tissue, which encompass various pathways and molecular mechanisms. Throughout this phase, ischemia causes ongoing cellular damage, which includes apoptosis resulting from alterations in the balance of Bcl-2 family proteins (e.g., Bax, Bcl-2, Bak) and cell death stemming from the release of cellular contents due to cellular injury (Tsujimoto, 1998; Iadecola, 2004; Rong and Distelhorst, 2008; Brenner and Mak, 2009; Pradelli et al., 2010; Lindsay et al., 2011; Estaquier et al., 2012). Mitochondrial dysfunction resulting from ischemia enhances the production of ROS, including superoxide anions and hydrogen peroxide. ROS induces cellular damage through oxidation of biological macromolecules, including lipids, proteins, and DNA. Oxidative stress may impair the activity of the antioxidant enzyme system, including superoxide dismutase (SOD), glutathione peroxidase (GPx), and catalase (CAT), thereby hindering the effective removal of ROS (Iadecola, 2004). The NF-kB signaling pathway is integral to this process. During the acute phase, impairment of the BBB facilitates the entry of blood-borne substances into brain tissue, worsening edema and tissue damage (Iadecola, 2004; Candelario-Jalil, 2009; Iadecola and Anrather, 2011; Sun and Nan, 2016). Activation of matrix metalloproteinases (MMPs) results in degradation of tight junction proteins (Furuse, 2010; Gupta and Ryan, 2010; Jin et al., 2013; Morrison and Filosa, 2013), thereby compromising the BBB.

In the subacute phase, the body’s protective functions are activated. The phosphoinositide 3-kinase (PI3K)/protein kinase B (AKT) pathway is involved in apoptotic signaling and provides protection during IS (Yang et al., 2019a; Diao et al., 2020), influencing cell survival and apoptosis through downstream targets, including NF-κB, mammalian target of rapamycin (mTOR), glycogen synthase kinase 3 beta (GSK-3β), and NOS. Inhibition of GSK-3β reduces apoptosis, whereas activation of Nrf2 enhances antioxidant protein expression and mitigates oxidative stress (Wang et al., 2022). IS is followed by an increase in VEGF expression. This increase facilitates the proliferation, migration, and lumen formation of vascular endothelial cells through receptor binding (Chen et al., 2019; **Figure 1**). The VEGF signaling pathway promotes vasodilation and maturation of new blood vessels by regulating downstream effectors, including endothelial NOS (eNOS), which produces nitric oxide (NO) (Zhang et al., 2000). The expression of neurotrophic factors, such as brain-derived neurotrophic factor (BDNF) and nerve growth factor (NGF), is also elevated. BDNF facilitates nerve regeneration and repair through activation of its receptor tropomyosin receptor kinase B (TrkB) (Wang et al., 2011), whereas NGF plays a role in neuroprotection through its receptor p75NTR (Mateo et al., 2007). Ischemia induces heightened autophagy, which partially facilitates the removal of damaged organelles and protein aggregates (Zhang et al., 2022). This activity is evidenced by elevated expression of LC3-II and an increased LC3-II/LC3-I ratio.

#### Chronic phase

The chronic phase of IS is a prolonged process that extends for weeks, months, or even years following the occurrence of the stroke (Bernhardt et al., 2017). The chronic inflammatory response and closure of the BBB are critical factors in brain injury and repair (Samal and Segura, 2021), complicating drug delivery. During the chronic phase, microglia and macrophages remain active, secreting pro-inflammatory cytokines such as TNF-α, IL-1β, and IL-6. These factors can activate inflammatory signaling pathways, such as the NF-κB pathway, resulting in the persistence and amplification of the inflammatory response. Chronic neuroinflammation may facilitate neurodegeneration, leading to cognitive decline and dementia. Neuronal degeneration can manifest in distant brain regions weeks to months following a stroke (McGeer and McGeer, 2004; Ran et al., 2021b). The closure of the BBB complicates drug delivery. These conditions hinder the regeneration of synaptic circuits, resulting in cognitive decline and potential neuronal apoptosis. Regulating the brain microenvironment during the chronic phase is essential for neuroprotection and neural repair.

### Pathological characteristics and treatment requirements across various phases of hemorrhagic stroke

#### Initial phase

**Figure 2:**
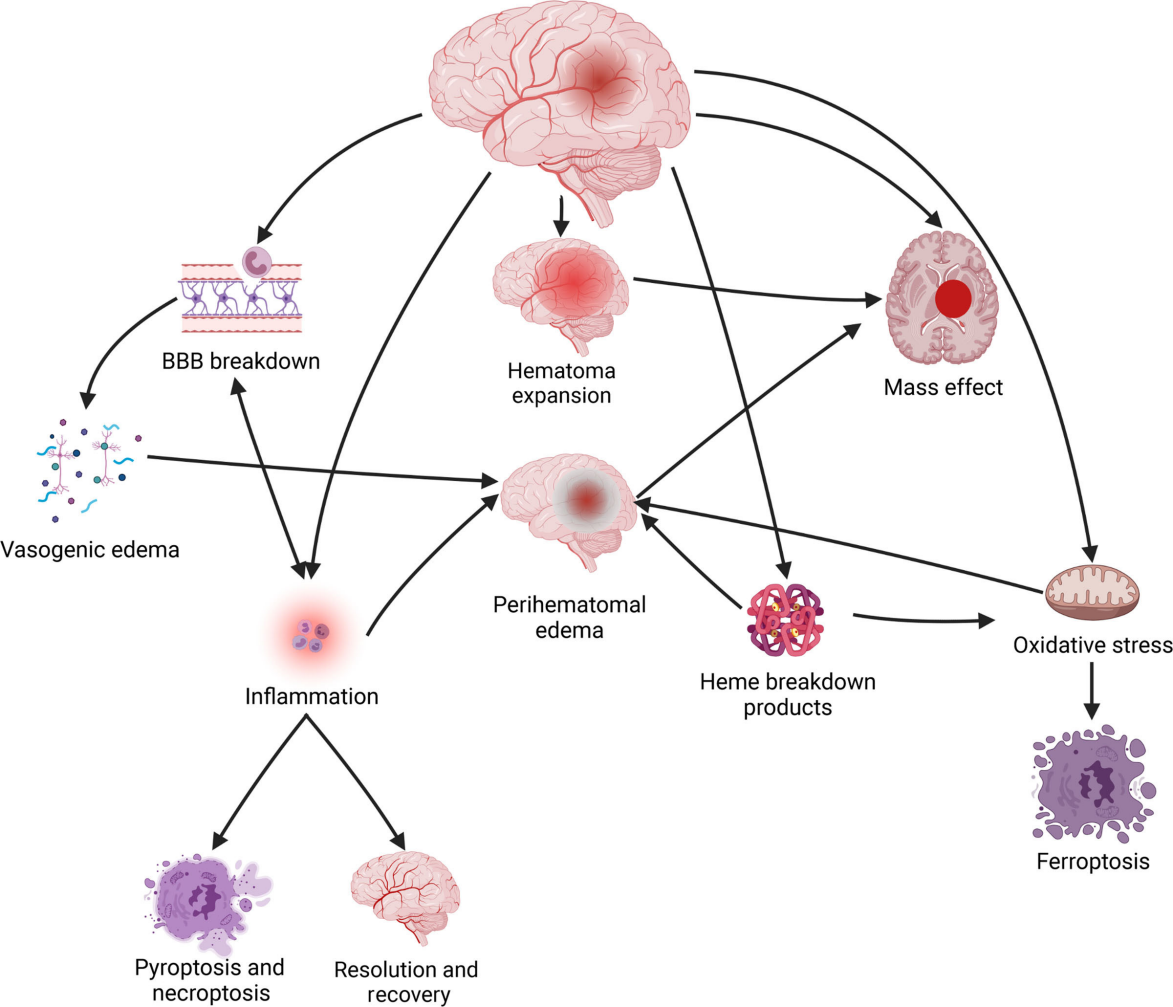
Mechanisms of injury after intracerebral hemorrhage. Reproduced with permission from Magid-Bernstein et al. (2022). BBB: Blood–brain barrier.

This phase pertains to the immediate period within 6 hours following the onset of intracerebral hemorrhage, during which the damage is primarily inflicted by the hemorrhage. Infiltration of extravasated blood into the brain parenchyma or subarachnoid space is accompanied by an immediate increase in intracranial pressure, potentially leading to arterial compression and subsequent cerebral ischemia. Concurrently, the mass effect of hematoma formation may result in the displacement of the brain’s midline and even the bulging and compression of critical structures in the brainstem (Anderson et al., 2010; Arima et al., 2010, 2012; Steiner and Bösel, 2010; Chaturvedi and Greenberg, 2011; Dowlatshahi et al., 2011; Yang et al., 2015; Magid-Bernstein et al., 2022; Xu et al., 2022b). The focus during this stage is to effectively manage the bleeding and mitigate intracranial pressure (**Figure 2**).

#### Acute and subacute phases

These two phases occur within a timeframe of 6 hours to 2 weeks following intracerebral hemorrhage. Due to the challenges in distinctly separating the pathological changes in these stages, they are addressed collectively. This phase of primary injury results in hematomas and edema, potentially leading to more significant secondary injury (Selim and Norton, 2020; Shi et al., 2021; Jiang et al., 2022). In the initial 24 to 72 hours, serum proteins permeate the adjacent parenchyma, resulting in angioedema. Over the next 7 to 14 days, key components in the blood, including thrombin, hemoglobin, and associated degradation products, along with neuroinflammation following HS and degradation products of vasoactive substances, contribute to the impairment of the BBB (**Figure 2**). This damage to the BBB subsequently intensifies the infiltration of inflammation within the brain (McCourt et al., 2015; Yang et al., 2023). In this series of reactions, MMPs generated by resident microglia, astrocytes, and endothelial cells within the central nervous system facilitate the degradation of the BBB and the upregulation of cell-adhesion molecules in the vicinity of the hematoma (Sanmarco et al., 2021; Lee et al., 2022; Lauzier and Athiraman, 2024; Liu et al., 2024). Additionally, they contribute to the production of additional pro-inflammatory factors via the NF-κB/TLR4 pathway (Ohashi et al., 2023; Gu et al., 2024). Thus, at this phase, obstructing this pathway may serve as an effective treatment (Devanney et al., 2020; Xu et al., 2020). Simultaneously, the oxidative stress resulting from the breakdown products of red blood cells, including iron ions and heme, along with mitochondrial dysfunction and the disruption of the antioxidant system following an HS, can contribute to cell apoptosis. Research indicates that the inhibition of iron-induced apoptosis may lead to a decrease in cell death surrounding the hematoma (Cui et al., 2021; Feng et al., 2021b; Shen et al., 2022; Zheng et al., 2022; Benarroch, 2023; Guo et al., 2023; Kenkhuis et al., 2023; Ryan et al., 2023; Hu et al., 2024).

#### Chronic phase

This phase occurs between 3 weeks and 6 months following bleeding, with the pathological changes during this timeframe primarily involving tissue repair and remodeling (Chen et al., 2014; Shi et al., 2019b; Magid-Bernstein et al., 2022). Glial cells within brain tissue undergo proliferation and contribute to the formation of glial scars. The prolonged presence of an occupying entity results in localized or widespread brain atrophy, which is accompanied by compensatory expansion of the brain ventricles. Inflammatory mediators, including interleukins and tumor necrosis factors, play a significant role in the chronic phase. Inflammatory reactions can be exacerbated, resulting in additional damage to brain tissue, and may also influence the survival and functional recovery of nerve cells (Lan et al., 2017; Shi et al., 2019a; Li et al., 2022b, 2024). The primary objective of treatment at this stage is to eliminate chronic inflammation and reorganize nerve synapses.

## Applications of Nanomedicines in Stroke

### Applications of nanomedicines in ischemic stroke

#### Extracellular vesicles

Extracellular vesicles (EVs) include exosomes, which are generally 30–150 nm in diameter; microvesicles, which typically range from 100 nm to 1 µm in diameter; and apoptosomes, which are usually greater than 1 µm in diameter (Yang et al., 2025). Exosomes have emerged as a promising drug-delivery system in nanomedicine because of their unique biological properties and significant role in intercellular communication. The benefits of EVs as drug carriers include low immunogenicity, high stability, biocompatibility, and the capacity to shield drug molecules from degradation in the *in vivo* environment (Herrmann et al., 2021; Lu et al., 2024). These characteristics render EVs a viable option for drug-delivery systems, particularly in the context of IS therapy.

EVs play an important role in IS by facilitating the transport of molecular cargo and modulating the associated immune cell activity. They transport protein cargo essential for intercellular communication and immune regulation (Zhou et al., 2020; Herrmann et al., 2021; Tian et al., 2021; N V Lakshmi Kavya et al., 2022; Lu et al., 2024). Proteins can function as signal molecules, surface receptors, enzymes, or cytokines, including growth factor receptors within signal transduction proteins, such as epidermal growth factor receptors (EGFRs) and vascular endothelial growth factor receptors (VEGFRs). These proteins activate signal transduction pathways in receptor cells, including the mitogen-activated protein kinase (MAPK), PI3K/AKT, and NF-κB pathways, thereby regulating cell proliferation, differentiation, and survival. Immune proteins significantly influence immune cell function, facilitating T cell activation through costimulatory signals or modulating the immune response through regulatory T cells. Surface and adhesion molecules, such as selectins, integrins, and immunoglobulin superfamily members, facilitate interaction and adhesion, thereby mediating the targeting and endocytosis of EVs. Tian et al. (2021) enhanced the targeting of EVs in the ischemic brain by isolating EVs from a human neural progenitor cell line and conjugating them with a recombinant fusion protein that included an RGD peptide. This approach successfully inhibited the inflammatory response following reperfusion in IS. In addition to their direct effects on the treatment of IS, EVs also influence the condition indirectly. Dong et al. (2019) isolated EVs from differentiated HL-60 cells. Their surface proteins exhibited high expression of integrin β2 and P-selectin glycoprotein ligand-1 (PSGL-1), facilitating targeting of the ischemic inflammatory region. These EVs demonstrated drug-loading capacity for targeted delivery of the anti-inflammatory mediator Resolvin D2 (RvD2) to sites of inflammation. This strategy facilitated prolonged drug release in the lesion area, thereby achieving a sustained therapeutic effect.

Previous research has highlighted multiple challenges in the clinical translation of EV-based therapies (Tian et al., 2021; Lu et al., 2024). The first factor is the efficiency of drug loading. The presence of residual materials from parent cells in EVs restricts the capacity for loading exogenous drugs into these vesicles. Additionally, the population of EVs is heterogeneous in size, composition, and surface markers, which can influence drug loading and release. Factors such as solubility, molecular size, charge characteristics, and interactions with the EV membrane can also affect loading efficiency. The other challenges are related to production and purification. The population of EVs exhibits heterogeneity in terms of size, protein, and lipid composition. Current isolation techniques, including ultracentrifugation, filtration, and density gradient centrifugation, may not effectively differentiate EVs from other biomolecules, such as protein aggregates and viruses. Furthermore, the complex composition and functionality of EVs complicate the establishment of standardized protocols for their isolation, purification, cryopreservation, and transportation. Related issues include quality control, safety and efficacy testing, and cost-effectiveness, all of which influence the application and translation of EVs.

#### Liposome-based nanomedicines

Liposomes are artificial membrane structures classified into small unilamellar vesicles, large unilamellar vesicles, multilamellar vesicles, and multi-vesicular liposomes on the basis of their architecture. Their primary components consist of phospholipids, cholesterol, and various additives, exhibiting a bilayer structure similar to that of biological membranes. Phospholipids are the main chemical components of liposomes, while cholesterol plays a crucial role in regulating membrane fluidity and permeability, enhancing liposome stability, and decreasing clearance rates in the body (Filipczak et al., 2020). During preparation, additives such as polyethylene glycol (PEG) derivatives are incorporated to enhance the long-circulation properties of liposomes, along with specific surface modifiers to improve targeting capabilities. These additives can improve liposome performance by extending the circulation time, increasing drug encapsulation efficiency, and enhancing targeting for specific cells. In comparison with other nanomedicines, liposomes exhibit high biocompatibility due to their structural similarity to biological membranes. They can be enzymatically degraded in the body, thereby reducing the potential risk of long-term accumulation. These vectors demonstrate favorable biodegradability and, as non-viral entities, exhibit reduced immunogenicity. These characteristics render liposomes suitable for application in the sensitive brain environment following IS.

Zhao et al. (2016b) and Sabry et al. (2023) addressed issues related to low drug-delivery efficiency across the BBB by administering nanodrugs intranasally to the affected regions of the brain. Zhao et al. (2016b) developed basic fibroblast growth factor for delivery to the brain through intranasally administered nanoliposomes, potentially improving post-stroke neuroprotection by facilitating angiogenesis and nerve regeneration. Basic fibroblast growth factor functions as a growth factor by binding to cell surface receptors, thereby activating downstream signaling pathways, including the PI3-K/Akt pathway, in the brain. This mechanism may enhance neuroprotection following a stroke by promoting angiogenesis and nerve regeneration.

Eissa et al. (2023) developed an intranasal delivery system utilizing solid lipid nanoparticles to enhance the bioavailability of valsartan in the brain, decrease the required dosage and frequency of administration, and increase patient compliance. Additionally, Zhao et al. (2016a) developed a dual-targeting system that uses two distinct peptides: the T7 peptide and the stroke homing peptide (SHp). The T7 peptide specifically binds to the transferrin receptor (TfR) on brain capillary endothelial cells, allowing the nanodrug to cross the BBB. SHp is a peptide identified through *in vivo* phage display that demonstrates a preference for localization in ischemic brain tissue, enabling precise localization of the drug to the ischemic region. This nanomedicine includes a neuroprotective agent, ZL006, which selectively inhibits the interaction between ischemia-induced neuronal NOS and postsynaptic density protein 95 kDa. These designs have the potential to enhance recovery from local cerebral ischemic injury in the context of middle cerebral artery occlusion (MCAO) and subsequent reperfusion conditions.

Nevertheless, liposomal nanomedicines present certain challenges. The primary concerns are related to stability. Liposomes can experience stability issues during circulation within the body, including alterations in particle size, morphological changes, and drug leakage. Additionally, liposomal nanomedicines may elicit immune responses in the body, which can influence their efficacy and safety. The production process for liposomal nanomedicines is often more complex and expensive than those for traditional pharmaceuticals. Although liposomal nanomedicines have demonstrated delivery potential in animal models and *in vitro* studies, numerous challenges remain to be addressed before these findings can be applied clinically.

#### Biomembrane-based nanomedicines

**Figure 3:**
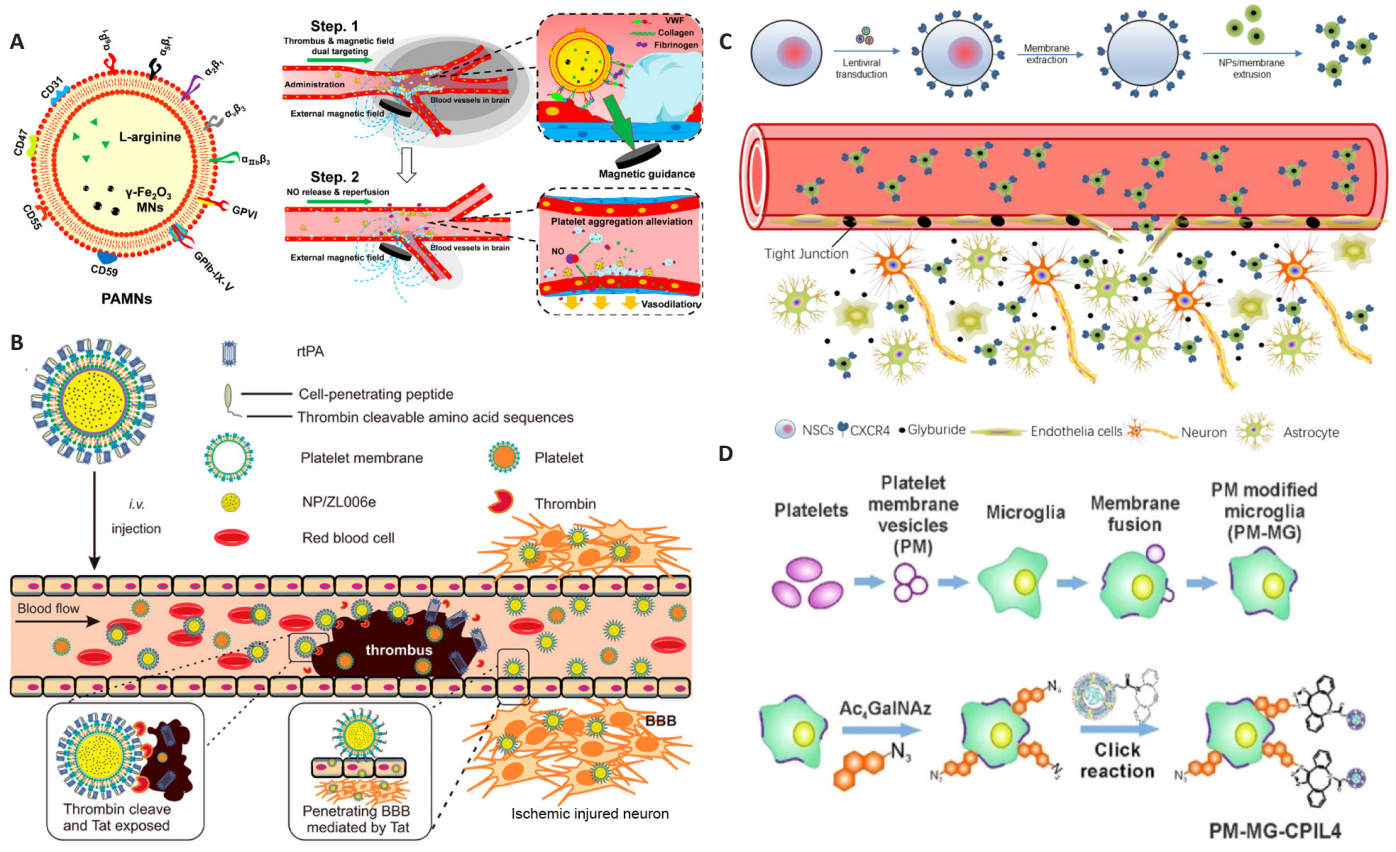
Application of biomembrane-based nanomedicines in ischemic stroke. (A) Platelet membrane nanomedicine, using the characteristics of platelets to target adhesion to damaged blood vessels (Li et al., 2020). (B) Platelet membrane nanomedicine, using the characteristics of platelets to escape phagocytosis by mononuclear macrophages in the circulatory system and target thrombi (Xu et al., 2019). (C) Neural stem cell membrane nanomedicine, modified by the neural stem cell membrane to enhance targeting of the ischemic brain region (Ma et al., 2019). (D) Platelet membrane nanomedicine, using the platelet membrane to enhance targeting of the drug to damaged blood vessels (Li et al., 2021c). Reproduced with permission from Xu et al., 2019 and Li et al., 2020. CXCR4: C–X–C motif chemokine receptor 4; NSCs: neural stem cells.

Biomembrane-based nanomedicines represent an innovative class of drug carriers. The primary categories of biomembranes used in such nanomedicines include red blood cell membranes, platelet membranes, white blood cell membranes, stem cell membranes, endoplasmic reticulum membranes, and composite biofilms (**Figure 3**). These nanomedicines replicate the structure and function of biological membranes to enhance drug-delivery efficiency and therapeutic outcomes (Xu et al., 2019; Li et al., 2021a, b, c, d). They exhibit low immunogenicity, high targeting capabilities, and favorable biocompatibility. Their ability to evade clearance by the reticuloendothelial system enables prolonged circulation within the body, enhancing drug efficacy and minimizing side effects. Macrophage membrane-like nanomedicines can extend a drug’s half-life by avoiding immune clearance in the circulatory system. They can also regulate the polarization state of macrophages in the ischemic infarct area, transitioning from the pro-inflammatory M1 type to the anti-inflammatory M2 type, and thereby reducing the inflammatory response. The presence of CD47 protein on the erythrocyte membrane can diminish drug clearance by the reticuloendothelial system, thereby extending circulation time within the body. Additionally, mature red blood cells lack nuclei and complex organelles, facilitating the extraction and purification of the red blood cell membrane. Ma et al. (2019) utilized nanomedicines coated with cell membranes derived from neural stem cells, which improved the migration of the drug to the ischemic brain area by leveraging the chemotactic properties of neural stem cells in that region. Dong et al. (2019) developed nanoparticle drugs derived from neutrophil membranes that specifically target inflammatory sites in the brain by exploiting the natural interaction between neutrophils and vascular endothelial cells. Some research has demonstrated that platelet membrane-like nanoparticle drugs can specifically target and adhere to damaged blood vessels (Xu et al., 2019; Li et al., 2020, 2021c). By loading various drugs, these nanoparticles can be utilized for the treatment of IS. Coating cell membrane drugs with cell membranes may diminish the initial immune response; however, the prolonged presence of nanoparticles in the body could trigger the complement system, induce antibody production, and result in chronic inflammatory or foreign body reactions. Simultaneously, achieving precise control of drug release within a specific microenvironment presents a significant challenge. Moreover, the engineering of nanomedicines presents ongoing challenges that must be addressed in the subsequent phase of their development.

#### Nanozymes

The use of nanozymes in the treatment of IS is a cutting-edge area of research (Chen et al., 2024; Yang et al., 2024). Nanozymes are a category of nanomaterials that exhibit catalytic activity similar to that of enzymes. They can replicate the functions of natural enzymes, facilitate the conversion of specific substrates, and display catalytic properties comparable to those of natural enzymes. Thus, the use of nanozymes represents a novel therapeutic alternative following IS. The investigation and utilization of nanozymes in IS are a significant focus at the intersection of nanotechnology and rehabilitation medicine.

The primary mechanisms for treating IS with nanoenzymes can be summarized as follows. Nanoenzymes eliminate ROS by mimicking the functions of several enzymes: SOD, catalase, glutathione peroxidase, and peroxidase (Wang et al., 2023; Singh, 2024; Yang et al., 2024). High levels of ROS in the ischemic infarct zone can be reduced by mimicking the activity of these enzymes. Decreasing oxidative stress effectively suppresses the synthesis of inflammatory mediators. Certain nanoenzymes utilize specific receptors that are overexpressed on the surface of endothelial cells in the BBB, including the TfR. Binding the nanoenzyme to transferrin enhances its affinity for TfR, facilitating receptor-mediated transport across the BBB and significantly improving drug-delivery efficiency in the middle and late phases of IS. The current applications of nanozymes are summarized in **Additional Table 2**.

**Additional Table 2:** Nanozyme-related applications in ischemic stroke

Nanozyme	Animal models	Administration	Dose	Therapeutic mechanism	Reference
PBzyme	MCAO (mice), male, 8-12 wk	Tail vein injection (1 h after surgery)	20 mg/kg	Promoting microglial polarization to M2 type; reducing caspase-3 activation; increasing expression of Bcl-2; reducing Bax expression	Liu et al., 2023a
PB@PDA NPs	MCAO (mice), HI (newborn mice), 7 d	Tail vein injection (mice); intracardiac injection (newborn mice) (2 h after surgery)	20 mg/kg (newborn mice)	Maintaining mitochondrial membrane potential; reducing caspase-3 activation; decreasing expression of pro-inflammatory cytokines	Zhao et al., 2024
HPBZs	MCAO (rat), male	Intracerebroventricular injection (1 h after surgery)	1.6 mg/kg	Inhibiting the activation of NF-kB; increasing expression of Bcl-2	Zhang et al., 2019
Fe_3_O_4_	MCAO (mice), male 8 wk	Oral administration; (3 d before surgery) intracerebroventricular injection	50 mg/kg (p.o.) 1.5 mg/kg, 3 mg/kg	Reducing immunoreactivity of GFAP; reducing MDA levels; increasing the expression of PECAM-1; increasing expression of ZO-1 and Claudin-5	Yan et al., 2021
Eda-MnO_2_@Tf	MCAO (rat), male	Tail vein injection (1 h after surgery)	0.4 mg/kg 0.6 mg/kg 0.8 mg/kg	Monitoring treatment; reducing levels of inflammatory factors (TNF-α, IL-1β, IL-6)	Zhao et al., 2022
MPBzyme@ NCM	tMCAO (mice), male 8-12 wk	Tail vein injection (1 h after surgery)	20 mg/kg	Activating the STAT6 pathway	Feng et al., 2021a
pDA-MNOF	MCAO (mice)	Intracerebroventricular injection (during surgery)		Activating the JAK-STAT3 pathway; increasing the expression of VEGF; reducing the volume of the stroke cavity	Wang et al., 2023

This table summarizes experimental investigations on nanomedicines for stroke, detailing the experimental model, delivery technique, dosage, and mechanism of therapeutic action. Findings from this table will serve as a reference for future studies on this category of medication. Bax: Bcl-2 associated X protein; Bcl-2: B-cell lymphoma 2; caspase-3: cysteine-dependent aspartate-specific protease-3; Eda-MnO_2_@Tf: transferrin-enabled blood-brain barrier crossing manganese-based nanozyme; GFAP: glial fibrillary acidic protein; HPBZs: hollow prussian blue nanozymes; ICAM-1: intercellular adhesion molecule 1; IL-1β: interleukin-1-beta; IL-6: interleukin-6; JAK-STAT3: Janus kinase-signal transducers and activators of transcription 3; MCAO: middle cerebral artery occlusion; MDA: malondialdehyde; MPBzyme@NCM: neutrophil-like cell-membrane-coated mesoporous Prussian blue nanozyme; NF-kB: nuclear factor kappa-light-chain-enhancer of activated B cells; PB@PDA NPs: polydopamine-coated Prussian blue nanoparticles; PBzyme: Prussian blue nanozyme; pDA-MNOF: polydopamine-modified manganese-organic framework; PECAM-1: platelet endothelial cell adhesion molecule-1; p.o.: postoperative; STAT6: signal transducer and activator of transcription 6; tMCAO: transient middle cerebral artery occlusion; TNF-α: tumor necrosis factor-alpha; VCAM-1: vascular cell adhesion molecule 1; VEGF: vascular endothelial growth factor; ZO-1: zonula occludens-1.

Wang et al. (2024) developed a peptide-templated manganese dioxide nanozyme (PNzyme/MnO_2_) that integrates the thrombolytic properties of the peptide chain with the ROS-scavenging capabilities of the nanozyme. This approach targets thrombi via a specific peptide chain sequence, initiating thrombolytic activity through the activation of thrombin within the thrombus. Furthermore, it effectively inhibits astrocyte activation and the secretion of pro-inflammatory cytokines, thus offering neuroprotection. Liao et al. (2024) developed cerium-based nanoenzymes (ceria nanoenzymes, CeNZs) that replicate the functions of SOD and catalase. These nanoenzymes effectively scavenge superoxide anions, hydrogen peroxide, and hydroxyl radicals, thereby safeguarding mitochondria from oxidative damage. This action inhibits the NF-κB pathway, decreases the release of pro-inflammatory factors, and promotes microglial polarization toward the M2 phenotype, contributing to anti-inflammatory responses and neuroprotection. Certain nanozymes can be engineered to attach to nanovesicles resembling cell membranes. Feng et al. (2021a) developed a Prussian blue nanozyme (MPBzyme) coated with a neutrophil membrane, resulting in the novel nanozyme MPBzyme@NCM. MPBzyme@NCM utilized the targeting properties of the neutrophil membrane to increase the accumulation of the nanozyme in damaged brain regions through interactions with post-inflammatory brain microvascular endothelial cells. MPBzyme@NCM reduced brain tissue damage and enhanced neurological function by leveraging the anti-inflammatory and neuroprotective properties of MPBzyme. The targeting of neutrophil membranes increased the accumulation of nanoenzymes in injured brain regions via interactions with post-inflammatory brain microvascular endothelial cells. MPBzyme exhibited anti-inflammatory and neuroprotective effects that mitigated brain tissue damage, enhanced neurological function, and elevated survival rates following IS.

The field of nanoenzymes faces two major issues at present. First, the specific mechanisms of action of nanoenzymes in the body, particularly their interactions with biomolecules and effects on intracellular signaling pathways, require further investigation (Singh, 2024; Yang et al., 2024). Second, a prevalent concern regarding many nanodrugs is their biocompatibility. Many nanozymes incorporate inorganic metal components, and the metabolism of these components upon entering the body presents a significant challenge. Consequently, the next step involves evaluating the removal of inorganic metal components from nanozymes while preserving their inherent activities to enhance clinical applicability.

#### Polymer nanomedicines

Polymer nanomedicines involve the application of nanotechnology to formulate nanoparticles of active pharmaceutical ingredients (APIs) and other substances or to integrate them with suitable carrier materials, resulting in effective drug formulations. Unlike conventional drugs, polymer nanomedicines are smaller and have a significantly higher surface area-to-volume ratio, which enhances their efficacy in drug delivery. Their surfaces can be chemically modified to provide excellent biocompatibility, stability, and the ability to target specific cells or tissues (Lim et al., 2019). Additionally, polymer nanomedicines can be engineered to respond to specific biochemical signals, including pH, temperature, and enzyme activity (Lv et al., 2018), and many of these nanomedicines are biodegradable. These unique characteristics underscore the potential of polymer nanomedicines for clinical translation.

Polymeric nanomedicines offer various therapeutic strategies for managing IS. Research has improved the *in vivo* application of natural drugs through re-modification, notably with compounds such as gallic acid and resveratrol (Lu et al., 2020; Zhao et al., 2020). Gallic acid, in particular, has been shown to significantly decrease nerve cell apoptosis and enhance the activity of antioxidant enzymes, including SOD and GSH-Px, while simultaneously reducing the levels of inflammatory factors such as TNF-α and IL-1β. Another strategy modifies the surface of the nanomedicine by applying an appropriate cell coating. For example, Ma et al. (2019) used neural stem cells that overexpress CXCR4 as an external coating for the drug, which enhanced the accumulation of the nanomedicine in the ischemic region of the brain. These strategies can be integrated on the basis of varying requirements to design and develop novel nanomedicines tailored to specific treatment needs.

Nevertheless, these medications present certain challenges. Polymer nanomedicines often exhibit low bioavailability due to inadequate water solubility, which constrains their clinical application. Additionally, the lack of dynamic monitoring of the brain microenvironment necessitates further investigation into the consistent environmental suitability of certain ROS- or pH-responsive drugs. In this regard, research should focus on identifying methods that can ensure selective accumulation of nanoparticles in lesion areas while preserving the integrity of healthy tissue.

#### Inorganic non-metallic nanomedicines

Inorganic non-metallic nanomedicines are a category of nanoscale pharmaceuticals characterized by distinctive physicochemical features and demonstrating considerable promise in the biomedical field, particularly in drug delivery and the treatment of critical neurological disorders. Iron oxide magnetic nanoparticles, for example, can be used for magnetic resonance imaging (MRI) and magnetically directed medication-delivery systems (Wu et al., 2020; Lu et al., 2021). In comparison with conventional pharmaceuticals, these nanoparticles offer unmatched magnetic targeting capabilities. Inorganic non-metallic nanomedicines can also be engineered as multifunctional platforms that integrate diagnosis, treatment, and targeting, thus realizing the concept of “theranostics.” Their unique attributes render them a prominent area of research for the treatment and diagnosis of IS. Certain inorganic metal nanomedicines exhibit distinctive antioxidant properties. Balasubramanian et al. (2020) and Li et al. (2022c) used cerium oxide nanoparticles as antioxidants to protect mitochondria by neutralizing ROS, thereby preserving their structure and function while reducing mitochondrial-mediated apoptosis and oxidative stress. Similarly, Li et al. (2021a) engineered manganese dioxide (MnO_2_) nanoparticles to mitigate oxidative stress by degrading excess H_2_O_2_ and converting it to O_2_. Inorganic non-metallic nanomedicines can also serve as detection instruments. Wu et al. (2020) labeled iron oxide nanoparticles on mesenchymal stem cells obtained from rats. The iron oxide nanoparticles enhanced the MRI visibility of the cells, enabling researchers to observe their dispersion and movement *in vivo*. This capability is crucial for understanding cellular function following treatment during the post-stroke rehabilitation phase. Moreover, associated nanomedicines have been utilized in fundamental research on IS. Li et al. (2021b) developed a magnetic targeting system using iron oxide (Fe_3_O_4_) and thrombin to replicate thrombosis in a stroke model, providing a robust tool for investigating stroke treatment and thrombolytic agents.

Despite the numerous advantages of inorganic metal nanomedicines, their utilization in IS is associated with several challenges and hurdles. First, metabolic concerns cannot be overlooked. The circulation and excretion mechanisms of inorganic non-metallic nanoparticles within the body remain inadequately understood. For example, Zhang et al. (2020a) demonstrated in a biodistribution study using nanoprobes that nanoparticles predominantly accumulate in the liver and spleen, indicating a potential risk of long-term accumulation. Furthermore, as inorganic non-metallic pharmaceuticals, these nanoparticles exhibit low immunogenicity and biocompatibility, necessitating surface modifications to enhance their interaction with relevant biological membranes, such as those of macrophages. This requirement raises both the cost and complexity of drug development.

#### Metallic nanomedicines

Metal nanomedicines are defined as nanomedicines utilizing metal nanoparticles for drug delivery or therapeutic purposes (Markman et al., 2013; Pan et al., 2022; Song et al., 2024). The surface of metal particles exhibits a high atomic density, facilitating modifications in comparison other materials. Targetability can be achieved through the attachment of various ligands. Certain metal particles exhibit distinctive optical properties, rendering them appropriate for photothermal therapy and biological imaging (Huang et al., 2007). Metals analogous to iron oxides exhibit magnetic properties and can serve as contrast agents in MRI or facilitate localized thermal effects in magnetic hyperthermia (Bulte et al., 2002). The characteristics of metal nanomedicines can enable their application in a wider range of diseases, including IS (Patra et al., 2018).

The use of metal nanomedicines has primarily focused on the diagnosis and treatment of IS. Wang et al. (2015) developed BaHoF5 nanoparticles to serve as high-performance contrast agents for CT imaging. These nanoparticles incorporated the high-atomic-number elements barium (Ba) and holmium (Ho), facilitating high-contrast imaging across various X-ray operating voltages for multimodal CT imaging applications, such as CT angiography (CTA) and CT perfusion (CTP) imaging and thereby enhancing the diagnostic capability for IS. These substances are metabolized by the liver, necessitating low doses and minimizing the risk of nephrotoxicity and other associated risks. Farr et al. (2014) developed core-shell silicon superparamagnetic sugar nanoparticles (SX@M nanomedicines) for MRI with the aim of detecting early endothelial inflammation following a stroke. Modification of sialic acid Lewis X (SX) enables specific binding to E and P selectins, which are highly expressed in endothelial cells during inflammatory processes. The superparamagnetism of nanomedicine enhances MRI contrast, facilitating imaging of inflamed areas. Hyun et al. (2013) immobilized fluorescein-labeled hyaluronic acid (HA) onto gold nanoparticles. In the presence of ROS, HA is released from the nanoparticle surface, leading to fluorescence recovery of fluorescein. This mechanism of energy transfer on the surface of nanoparticles quenches probe fluorescence in the absence of ROS and enhances fluorescence in their presence. Thus, this allows the detection of ROS in animal stroke models and facilitates the study of ROS distribution levels in the brain post-stroke.

The primary issue associated with the use of metal nanomedicines is their biocompatibility. Metal ions may exhibit toxicity to living organisms, potentially inducing oxidative stress in cells, which can impair cellular function and result in cell death (Oberdörster et al., 2005). The long-term accumulation and distribution of metal nanomedicines in the body remains inadequately understood. Stability issues also require consideration. The physiological environment in the body, including factors such as pH and ionic strength, can influence the stability of metal nanomedicines. This may result in aggregation, sedimentation, or the shedding of surface modifications of nanoparticles (Alexis et al., 2008), ultimately impacting their therapeutic efficacy.

### Applications of nanomedicines in hemorrhagic stroke

#### Liposome-based nanomedicines

Liposome-based nanomedicines have been investigated in the area of HS. Kim et al. (2014) developed echogenic liposomes loaded with NO (NO-ELIP), and ultrasound-activated NO-ELIP demonstrated the ability to promote *in vivo* arterial vasodilation in experiments conducted on rabbit carotid arteries, effectively addressing the severe ischemic neurological deficits resulting from delayed cerebral vasospasm following subarachnoid hemorrhage. IL-10 can enhance the recovery of HS; however, development of effective delivery methods for IL-10 is ongoing. Han et al. (2023) created a nanoparticle system utilizing phosphatidylserine (PS) liposomes (PSL) for the delivery of IL-10. PS demonstrates effective targeting of microglial cells. Administering IL-10 to the targeted region stimulates the activation of signal transducer and activator of transcription 3 (STAT3) in microglia/macrophages. It enhances CD36 expression, facilitating hematoma resolution and reducing inflammation, thereby improving outcomes during the acute phase of HS. Comparable designs feature the liposome siRNA/CUR@SLN developed by Abudurexiti et al. (2024) for the nasal delivery of curcumin and siRNA. Nasal delivery can effectively bypass the BBB, and by addressing inflammation through a non-injection method, it can mitigate the secondary damage associated with inflammation following hyperthermic stress. The liposome enhances the stability of curcumin and siRNA, ultimately demonstrating effective brain-targeting and anti-inflammatory properties.

#### Polymer nanomedicines

Polymer nanoparticles are among the most extensively utilized drug nanocarriers at present. Their benefits include low toxicity, excellent biocompatibility, biodegradability, a high encapsulation rate, and robust sustained-release capabilities (Grabnar and Kristl, 2011; Rani et al., 2015; El-Say and El-Sawy, 2017; Moya-Lopez et al., 2022). These nanoparticles have the potential to address HS by enhancing pharmacological properties or advancing neuroprotective agents and genes. Tian et al. (2021) developed nanomicelles that incorporate rosuvastatin. This statin medication can inhibit inflammatory responses, scavenge free radicals, and enhance endothelial function, making it a potential neuroprotective agent following cerebral hemorrhage (Satoh et al., 2015; Sabatine et al., 2017; Li et al., 2018; Zhang et al., 2020b). The study utilized polyethylene glycol-polycaprolactone copolymer (PEG-PCL) to address the challenges of poor absorption and low oral bioavailability resulting from limited water solubility. This intervention led to a reduction in neuronal degeneration, inhibited inflammatory infiltration, decreased cerebral edema, and enhanced neurological function following HS. The degradation of hemoglobin after hemorrhagic shock results in a significant release of iron, which can lead to iron-dependent programmed cell death in the affected edema region (Koury and Ponka, 2004; Stockwell et al., 2017). Yang et al. (2021) utilized the antioxidant and neuroprotective properties of curcumin to develop curcumin-based polymer nanoparticles for encapsulation, effectively mitigating iron-induced apoptosis by modulating the NRF2/HO1 pathway to enhance the expression of GPX4. Catechins, as natural polyphenols, possess the ability to scavenge active oxygen species and chelate metal ions (Musial et al., 2020; Farhan, 2022). Zeng et al. (2025) developed catechin-based polyphenol nanoparticles (CNPs@PEG), which are polyphenol nanoparticles modified with thiol-terminated polyethylene glycol, with the aim of providing a potential treatment for conditions associated with iron toxicity and lipid oxidation.

#### Metallic nanomedicines

Cerium oxide nanoparticles (Nelson et al., 2016; Hosseini and Mozafari, 2020; Saifi et al., 2021; Shcherbakov et al., 2021) have been utilized in various applications in IS, demonstrating antioxidant capacity that effectively eliminates ROS in the brain following a stroke. This property makes them suitable for application in HS. Jeong et al. (2018) used aminohexanoic acid as a surface modifier to enhance cerium oxide nanoparticles. The incorporation of aminohexanoic acid led to an increased Ce^3+^/Ce^4+^ ratio in the synthesized cerium oxide nanoparticles, enhancing their antioxidant capacity and biocompatibility, and thereby making them more appropriate for *in vivo* applications. This design effectively eliminated a significant quantity of ROS generated by subarachnoid hemorrhage, providing therapeutic benefits by directly mitigating oxidative damage to nerve cells resulting from ischemia and indirectly suppressing highly detrimental neuroinflammation.

#### Carbon-based nanomaterials

Carbon-based nanomaterials, including graphene, graphene oxide, carbon black nanoparticles, fullerenes, carbon nanotubes, and carbon nanofibers, have applications in disease treatment and diagnosis (Peng et al., 2020; Zhang et al., 2021; Díez-Pascual, 2024; Bai et al., 2025). Dharmalingam et al. (2020) integrated deferasirox (DEF) with polyethylene glycol-conjugated hydrophilic carbon clusters (PEG-HCC) to create multifunctional nanoparticles designated as DEF-HCC-PEGs. PEG-HCCs have the capability to convert superoxide anions into oxygen, thereby effectively scavenging free radicals and diminishing oxidative stress. DEF can efficiently eliminate intracellular iron ions and mitigate the oxidative damage associated with iron. The results for the experimental group demonstrated that DEF-HCC-PEGs effectively inhibit the toxicity associated with heme and iron, restore the integrity of both nuclear and mitochondrial genomes, and decrease the expression of cell senescence markers. These findings represent an innovative approach for the treatment of HS. While carbon-based nanomaterials exhibit significant promise in HS, they are also associated with hidden risks. Several studies (Yang et al., 2008; Erdely et al., 2009, 2011; Ema et al., 2016; Yuan et al., 2019) have demonstrated that carbon nanomaterials can activate immune cells, particularly macrophages, and elicit inflammatory responses. For instance, carbon nanotubes may be engulfed by macrophages, resulting in disruption of lysosomal membranes and the generation of ROS, subsequently initiating apoptosis. Carbon nanomaterials can induce inflammatory responses in the lungs and stimulate immune cells, including macrophages and lymphocytes, upon introduction into animal models through various pathways, such as inhalation or injection. These aspects require attention during the application process.

#### Self-assembled peptide hydrogels

**Figure 4:**
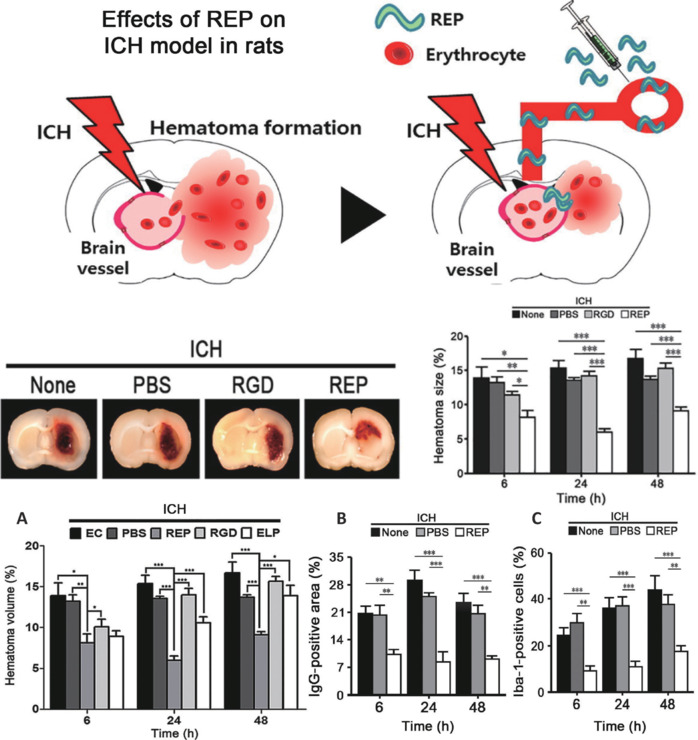
Utilization of self-assembled peptide hydrogel in ischemic stroke. ELP was modified to create a fusion protein that includes ELP (V140) and a RGD peptide, referred to as REP. This peptide has several functions, including reducing hematoma volume, occluding damaged blood vessels, and modulating inflammation. (A) The REP therapy group significantly reduced hematoma volume. Perivascular IgG can indicate leakage from damaged cerebral blood vessels. (B) The REP treatment group exhibited markedly lower IgG expression, suggesting that REP can inhibit the extravasation of blood components from damaged blood vessels into the brain parenchyma following cerebral hemorrhage. (C) Iba1 specifically identifies active microglial cells. The REP treatment group showed the lowest percentage of positive cells, indicating that REP administration enhances macrophage activation in the immune response following cerebral hemorrhage. Reproduced with permission from Park et al., 2017. ELP: Elastin-like polypeptide; Iba-1: ionized calcium-binding adapter molecule 1; PBS: phosphate buffered saline; REP: RGD-based elastin-like polypeptide; RGD: repeating integrin-binding arginine-glycine-aspartic acid.

Self-assembled peptide hydrogels are soft materials created through self-assembly of peptide molecules. Their structure consists of a three-dimensional fibrous network that is crosslinked through either physical or chemical bonds. These materials show substantial water content, a porous structure, adjustable mechanical stability, favorable biocompatibility, excellent injectability, and elasticity comparable to that of natural tissue. This makes them suitable for use as a drug-delivery vehicle for HS (Gelain et al., 2006, 2020; Liu et al., 2013; Alshehri et al., 2021). RADA16-I (Ac-RADARADARADARADA-CONH2) is a widely recognized self-assembling peptide (Loo et al., 2012; Cormier et al., 2013; Koutsopoulos, 2016; Liu et al., 2022) consisting of amino acids such as arginine (R), alanine (A), and aspartic acid (D). Sang et al. (2015) administered RADA16-I into the cerebral hemorrhage region following the aspiration of the hematoma, demonstrating its effectiveness in replacing the hematoma. This approach significantly diminished brain edema in the impacted lateral striatum and cortex while also decreasing the presence of neutrophils and macrophages in the affected region. The sensory-motor function of the rats in the treatment group showed significant improvement at 8 and 10 weeks post-surgery, and the volume of the brain cavity showed a notable reduction after the 10^th^ week. Elastin-like polypeptides (ELPs) are composed of repetitive pentapeptide sequences, typically (VPGXG), where V denotes valine, P indicates proline, G signifies glycine, and X represents a variable amino acid, commonly alanine or another hydrophobic amino acid (van Strien et al., 2023). This structure imparts distinctive physical and chemical properties to ELPs. ELPs demonstrate low critical solution temperature behavior, remaining soluble at lower temperatures while precipitating from the solution as the temperature rises. This property can enhance cell-adhesion capabilities (Varanko et al., 2020; Chen et al., 2021b; de Haas et al., 2023; Hong et al., 2024). Park et al. (2017) administered REP 3 hours post-molding. The treatment group demonstrated a notable decrease in hematoma volume at 6 hours (8.1%), 24 hours (6.0%), and 48 hours (9.1%), in contrast to the control group, which exhibited reductions of 13.9%, 15.4%, and 16.7%, respectively. REP also decreased immunoglobulin G leakage and reduced the expression of von Willebrand factor (**Figure 4**). The thermosensitivity, cell-adhesion capabilities, biocompatibility, and biodegradability of REP make it an outstanding nanomedicine with significant potential for various applications.

## Overall Evaluation of Nanomedicines

### Nanomedicine administration to the brain

**Figure 5:**
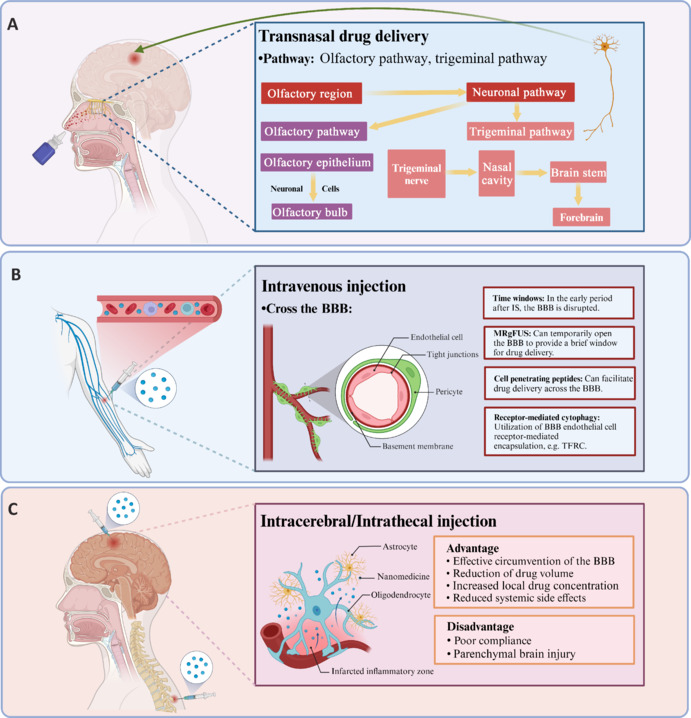
Delivery methodologies of nanomedicine. (A) Two pathways for transnasal medication administration. (B) Intravenous medication administration requires traversing the BBB. (C) Intracerebral injection and intrathecal injection. Created in BioRender. Erter, E. (2025). https://BioRender.com/r87y104. BBB: Blood–brain barrier; MRgFUS: magnetic resonance-guided focused ultrasound; TFRC: transferrin receptor.

The primary drug-delivery methods are intravenous, intranasal, *in situ*, intrathecal, deep cervical lymph node route administration, and oral administration (Ma et al., 2022; Liu et al., 2023c; Zhang et al., 2024a). The merits and demerits of different medication-delivery methods are outlined in the following sentences (**Additional Table 3** and **Figure 5**). The predominant drug-delivery methods in nanomedicine research for stroke treatment include intravenous, intranasal, and intrathecal injection. In comparison to other drug-delivery methods, these three are more convenient to administer. However, intrathecal injection, especially administration via the deep cervical lymph node route, can lead to major problems. Administration through the deep cervical lymph node pathway may result in localized inflammation, lymphadenopathy, or thrombosis. Moreover, intrathecal injection may induce fever, headache, neck rigidity, and cloudy cerebrospinal fluid. In extreme instances, it may cause critical complications such as meningitis and subdural abscess. Researchers should meticulously evaluate these issues throughout the design of experiments.

**Additional Table 3:** Advantages and disadvantages of different administration methods and their application

Delivery method	Advantage	Disadvantage	Reference
Deep cervical lymph node administration	Effectively avoid BBB; avoid first-pass effect; prolong action of drugs	Invasive; complex operation; potential complications	Qj et al., 2023
Intrathecal injection	Few systemic side effects; effective circumvention of the BBB; high local drug concentration	High operational requirements; susceptible to infection; immune response	Okuda et al., 2000; Krol et al., 2013
Intravascular injection	Rapid drug distribution; broad applicability	Invasive; susceptible to infection; susceptible to rapid clearance; affected by BBB	Li et al., 2021a, 2022c; Tian et al., 2021; Mu et al., 2023
Intravenous injection	Effectively avoid BBB; fast onset; precise targeting; high local drug concentration	Invasive; prone to infection; low compliance; complex operation	Hyun et al., 2013; Balasubramanian et al., 2020
Oral administration	Convenient drug administration; good cost-effectiveness; application of wide drugs	Low bioavailability	Yang et al., 2019b
Tranasal administration	Effective BBB penetration; non-invasive drug delivery; avoid first-pass effect; rapid absorption	Irritation of mucous membranes; limited absorption capacity; drug stability affected by intranasal microenvironment	Li et al., 2022a; Sabry et al., 2023

This table outlines the current administration techniques for experimental stroke therapies, along with their respective advantages and disadvantages. It serves as a resource for researchers to understand different administration methods and make informed decisions while designing trials. BBB: Blood-brain barrier.

The BBB is a selectively permeable barrier formed by tight junctions among the microvascular endothelial cells of the central nervous system. The BBB obstructs the entry of toxins and infections from the bloodstream into the brain while simultaneously hindering the administration of pharmaceuticals to the brain. Under normal circumstances, only water-soluble small molecules with molecular weight under 150 Da and hydrophobic small molecules with molecular weight below 400–600 Da can traverse the BBB through passive diffusion. The endothelial cells of the BBB contain various efflux pumps, including P-glycoprotein. Although certain medications can cross the BBB and penetrate the brain, the presence of efflux pumps reduces their duration of action within the brain. These pumps can actively transport drugs that have entered the cells back into the bloodstream, thereby decreasing the drug’s concentration in the brain and limiting its therapeutic efficacy. While some lipophilic pharmaceuticals are more likely to cross the BBB, enhancing a drug’s lipophilicity may diminish its biological efficacy. Moreover, highly lipophilic medications tend to exhibit prolonged retention in non-target organs and tissues, potentially leading to more severe adverse effects. These challenges make the BBB a significant obstacle for drug administration to the brain following a stroke (Wu et al., 2023). After a stroke, the brain microenvironment undergoes dynamic changes, with varying levels of ROS and pH across different brain regions (Dirnagl et al., 1999). Conventional medications often fall short in addressing these issues simultaneously (Chen et al., 2021a). Consequently, the surface functionalization of nanomedicines is of paramount importance, as elaborated below.

### Challenges and solutions in the administration of nanomedicines

As mentioned previously, the delivery of nanomedicines presents several challenges, for which multiple solutions have been proposed. These solutions are together referred to as surface functionalization of nanomedicines. We have summarized various surface-modification options in **Additional Table 4** and aim to provide further inspiration through these solutions.

**Additional Table 4:** Relevant surface modifiers and their functions in stroke

Surface modifier	Receptor	Capability	Reference
83-14 Mab, 29B4	Insulin receptor	Cross the BBB	Ulbrich et al., 2011; Kuo and Ko, 2013
Adenosine	Adenosine G-protein-coupled receptors A2	Increase BBB permeability; neuroprotective; anti-inflammatory; promote angiogenesis	Carman et al., 2011
Angiopep-2, apolipoprotein E peptide	LRP receptor	Cross the BBB	Demeule et al., 2008; Shao et al., 2010; Shen et al., 2011
Apolipoprotein chitosan CPPs	LDL receptor	Cross the BBB; high biocompatibility; increase BBB; permeability; anti-inflammatory	Neves et al., 2016 Saini et al., 2021 You et al., 2023
CRM197	Diphtheria toxin receptor	Cross the BBB	Tosi et al., 2015
DNA homologue-aptamer	Transferrin receptor	Increase BBB permeability	McConnell et al., 2019
Folic acid	Folate receptor	Increase drug concentration in the brain	Afzalipour et al., 2019
Glucose	GLUT receptor	Increase the BBB permeability; nutritional support	Min et al., 2020
Lactoferrin	LRP receptor and lactoferrin receptor	Reduce inflammation and apoptosis; increase BBB permeability	Luo et al., 2019
Leptin30 peptide	Leptin receptor	Cross the BBB	Jo et al., 2012
L-Glutathione	Glutathione receptor	Through the BBB; anti-oxidation	Englert et al., 2016
MG1 peptide	Ml microglia	Targeted at M1 microglia;	Yin et al., 2022
OX26, RI7217, 8D3	Transferrin receptor	Cross the BBB	Fornaguera et al., 2015; Johnsen et al., 2019
PEG		High biocompatibility; improve medication utilization	Santos et al., 2018
Peptide T7, peptide B6, THR,	Transferrin receptor	Cross the BBB	Prades et al., 2012; Zhao et al., 2016a
CGGGHKYLRW			
Polysorbate 80, poloxamer 188	LDL receptor	Improve drug stability; increase BBB permeability;	Kreuter, 2001; Mittal et al., 2011
Protamine		Increase BBB permeability; neuroprotective; anti-inflammatory	Kneer et al., 2023
SS-31 (Elamipretide)	Cardiolipin	Antioxidant; maintain mitochondrial function;anti-inflammatory; neuroprotective	Kim et al., 2015
Transferrin	Transferrin receptor	Cross the BBB	Yin et al., 2016

This table outlines the surface modifications of nanomedicines in stroke research, along with their target areas and functions. It helps researchers gain a clearer and faster understanding of these surface modifiers, thus assisting in the selection of appropriate modifiers for experimental design based on varying research needs. 29B4: Anti-insulin receptor β antibody; 83-14 Mab: anti-insulin receptor alpha antibody; 8D3: anti-mouse CD71/transferrin receptor antibody; BBB: blood-brain barrier; CGGGHKYLRW: transferrin-binding peptide; CPPs: cell-penetrating peptides; CRM197: cross-reacting material 197; GLUT: glucose transporter; LDL: low-density lipoprotein; LRP: low-density lipoprotein receptor-related protein; OX26: anti-transferrin receptor antibody; PEG: polyethylene glycol; RI7217: anti-CD71/transferrin receptor antibody; THR: threonine.

#### Surface functionalization and targeting of nanomedicines

Improving drug targeting can significantly enhance the efficacy of drugs. Certain targeting ligands on the surface of nanodrugs can be modified to improve drug delivery to necrotic or inflammatory cells and their associated organelles. Another approach involves coating the surfaces of nanodrugs with relevant cell membranes, such as using platelet membranes to direct drugs to injured cerebral blood vessels. Additionally, some nanomedicines are engineered to respond to specific microenvironments, including changes in pH or ROS (Koo et al., 2024). For instance, phenylboronic acid ester, poly(sulfur-containing polymer), ferrocene, anthocyanins, and response groups containing sulfur elements such as sulfides, sulfoxides, selenides, diselenides, and tellurides have been engineered into nanomedicines to trigger a ROS response in diseased areas (Matsui et al., 2014; Sobhani and Salavati-Niasari, 2021; Bierbaumer et al., 2022). Selenides, tellurides, and other sulfur-based response groups are utilized to elicit ROS and low pH responses in ischemic infarction regions by incorporating thiols, disulfide linkages, and polyelectrolyte polymer architectures into nanodrugs. These strategies can optimize drug targeting to maximize therapeutic efficacy. Lu et al. (2019) developed a liposomal dual-targeted nanomedicine (T7&SHp-P-LPs/ZL006) that effectively delivers neuroprotective agents to the lesion site by simultaneously targeting the BBB and ischemic infarction. Jian et al. (2018) administered the drugs into the infarct cavity during the subacute phase, causing a significant reduction in cavity volume due to the sustained-release properties of the polymer hydrogel. These effective treatments offer novel strategies for enhancing neuroplasticity, regulating inflammation, and facilitating vascular regeneration during the rehabilitation phase of stroke.

#### Biocompatibility of nanomedicines

In drug-delivery systems, the biocompatibility of nanoparticles is a critical criterion for their effective utilization as carrier molecules (Sundar et al., 2016). The physicochemical characteristics of nanoparticles, including size, surface shape, ζ potential, and surface chemistry, profoundly influence biocompatibility. The selection of particle size is critical; excessively small particles may result in inadequate medication stability within the body, increasing the likelihood of aggregation or depolymerization. Nanodrugs with excessively small particle sizes may be swiftly eliminated from the body via renal filtration, leading to a transient therapeutic effect. Additionally, small particle sizes may result in unintended biodistribution, potentially crossing tight biological barriers and causing non-specific toxicity. Conversely, nanoparticles with excessively large dimensions may be rapidly eliminated by the mononuclear phagocyte system, including macrophages in the liver and spleen, leading to a reduced duration of circulation. Nanoparticles with large particle sizes may also show diminished capacity to traverse biological barriers, such as the vascular wall and the BBB, thereby influencing the distribution and targeting of the medication in tissues (Dong et al., 2019; Tian et al., 2021). A previous study has indicated that nanoparticles between 50 and 200 nm in size demonstrate enhanced biocompatibility in therapeutic investigations for conditions such as stroke, Alzheimer’s disease, and Parkinson disease (Meng et al., 2022). In terms of nanodrug morphology, nanoparticles with rod and worm shapes have shown superior efficacy than spherical particles in penetrating cellular barriers and accessing the cell nucleus (Hinde et al., 2017).

A high absolute value of the zeta potential typically indicates enhanced dispersibility and a reduced propensity for aggregation, facilitating the diffusion of drug within the body. The positive and negative values of the zeta potential simultaneously influence the interaction between nanoparticles and the cell membrane (Bhattacharjee, 2016; Smith et al., 2017; Holsæter et al., 2022). Consequently, in nanodrug design, selection of drugs with charges opposite to those of the cell membrane in the target area are essential to enhance the affinity of drug. The modification of drug surfaces frequently involves the use of PEG to enhance the water solubility and stability of nanodrugs (D’Souza and Shegokar, 2016; Zhang et al., 2016; Song et al., 2023). Researchers have utilized platelet cell membranes, red blood cell membranes, and macrophage cell membranes, among others, for modification (Dong et al., 2019; Ma et al., 2019; Xu et al., 2019; Li et al., 2020, 2021a, b, c, d). In comparison to PEG, modification with cell membranes exhibits superior biosafety. In terms of therapeutic efficacy, a judicious focus on the biocompatibility of nanomedicines can enhance drug accumulation in the ischemic infarction region during the recovery phase of IS with a reduced dosage, thereby optimizing therapeutic outcomes. In future studies, researchers must focus more intently on the aforementioned challenges and further enhance the biocompatibility of nanomedicines.

#### Safety and toxicity of nanomedicines

Safety and toxicity are of utmost significance for nanomedicines, and this is the principal focus of research designs. The metabolic elimination of nanomedicines in the body primarily occurs in the liver and kidneys. The liver performs biotransformation primarily through phagocytosis by Kupffer cells and the enzymatic system, which includes cytochrome P450 enzymes. Conversely, the kidneys primarily achieve metabolic clearance through filtration. The dimensions and morphology of nanomedicines can influence their biodistribution, clearance rate, and cellular internalization. Smaller nanoparticles exhibit a higher propensity for cellular uptake and may possess cytotoxic properties. Research has demonstrated a disparity in the clearance patterns of organic and inorganic nanoparticles within the brain (Gao et al., 2024). Organic nanoparticles stimulate the extracellular signal-related kinase 1/2 (ERK1/2) signaling pathway, enhance the release of microglial EVs, and are processed via the perivascular glial lymphatic system. Conversely, inorganic nanoparticles obstruct this pathway and remain in the brain. Nanomedicines, including those based on metal or carbon, may accumulate in the brain, trigger inflammatory reactions in the body, and potentially induce dementia (Ransohoff, 2016; Hosoo et al., 2017; Wang et al., 2018). PEG modification is a frequently employed approach to address these challenges. Another strategy involves utilizing cell membrane nanodrugs to circumvent the body’s immune system. Certain studies have opted to use natural plant extracts to mitigate medication toxicity. Deng et al. (2019) developed a nanodrug utilizing betulinic acid, which yielded favorable outcomes. Mu et al. (2023) used ROS-responsive polymers and targeted peptides to modify *Chuanxiong* particles. Nanocarriers addressed the issue of the inadequate water solubility of *Chuanxiong* in its native state, enhanced its bioavailability, and diminished the biological toxicity of the drug. The predominant method for monitoring pharmaceuticals within the body involves the use of surface-modified fluorescent markers, which provide a visual representation of the drug’s status. Hematoxylin & eosin staining may serve as an ancillary detection method; nevertheless, research on the long-term effects of medicines on the body remains limited. Fang et al. (2023a) recently introduced the notion of full-API nanodrugs, which contain 100% active pharmaceutical ingredient content and thus substantially diminish the metabolic burden of nanodrugs in the body. Future studies on nanodrugs should involve the development of full-API drugs to reduce the metabolic burden of pharmaceuticals to the greatest extent feasible.

### Experimental models and clinical applications of nanomedicines

Numerous studies continue to use rodent models for efficacy evaluation. The model types can be broadly classified into three categories: global IS, focal IS, and intracerebral hemorrhage. **Additional Table 5** summarizes the specific models, along with their merits and limitations. In the reviewed studies, rats and mice were the predominant animal species utilized in research, while the number of investigations using pigs as subjects is minimal. Experiments involving primates, particularly rhesus monkeys, are also limited. In comparison with small mammals, primates offer therapeutic benefits that more closely align with clinical outcomes and are more readily translatable to clinical applications. The existing experimental treatments predominantly focus on the acute or subacute phases, with scant attention given to the therapeutic effects during the recovery period. This issue may require prioritization in forthcoming research endeavors.

**Additional Table 5:** Animal models of stroke

Model	Advantage	Disadvantage	Reference
**Global ischemic stroke**			
2-VO model	Low mortality; controllable recirculation	Poor reproducibility	Sanderson and Wider, 2013; Li and Zhang, 2021
4-VO model	Easy to prepare; high reproducibility; low incidence of seizures	Permanent occlusion of vertebral arteries; high mortality	Li and Zhang, 2021; Tuo et al., 2021; Kim et al., 2023
Complete global brain ischemia	Clinically similar	Low survival rate; complex operation; many complications	Li and Zhang, 2021
**Focal ischemic stroke**			
Artificial sphere occlusion	Reproductivity of macrosphere embolization	Poor reproducibility of microsphere models	Bremer et al., 1975; Watanabe et al., 1977; Sommer, 2017; Li and Zhang, 2021; Narayan et al., 2021
Endothelin-1 occlusion	Easy manipulation	Affected by anesthetics	Sommer, 2017; Li and Zhang, 2021; Narayan et al., 2021; Lin et al., 2022
Endovascular filament occlusion	Easy manipulation; controllable reperfusion; ischemic penumbra	Poor stability; not suitable for thrombolysis	Sutherland et al., 2016; Sommer, 2017; Li and Zhang, 2021; Narayan et al., 2021
Photothrombosis model	Easy manipulation; long-term survival; reproducibility	Lack of penumbra	Sommer, 2017; Li and Zhang, 2021; Narayan et al., 2021; Kalyuzhnaya et al., 2023, 2024
Thromboembolic occlusion	Investigate thrombolytic processes	Poor repeatability	Ansar et al., 2014; Chen et al., 2015; Sommer, 2017; Li and Zhang, 2021; Narayan et al., 2021
Transcranial occlusion	Small infarct volume; low mortality rate; high repeatability	Destroy dura; intracranial infection	Sommer, 2017; Li and Zhang, 2021; Narayan et al., 2021; Sheng et al., 2023
Collagenase-induced model of intracerebral hemorrhage	Easy manipulation; controllable size of hematoma	Slow and diffuse bleeding; exacerbated inflammatory response	Germonpré et al., 2020; Wilkinson et al., 2020; Li and Zhang, 2021; Fang et al., 2023b
Whole blood injection model	Mimic the hematoma mass effect and blood toxicity	Uncontrollable size of hematoma; not suitable for studying bleeding and hemostasi	Pedard et al., 2020; Ding et al., 2021; Jia et al., 2021; Li and s Zhang, 2021; Fang et al., 2023b

This table presents various animal models of stroke used in research, including global ischemic stroke, focal ischemic stroke, and intracerebral hemorrhage. It outlines the advantages and disadvantages of each type, helping researchers select the appropriate stroke model for their specific research objectives. 2-VO model: Two-vessel occlusion model; 4-VO model: four-vessel occlusion model.

Regarding the therapeutic application of nanomedicines, particularly in relation to clinical translation (**Additional Table 6**), all existing clinical studies have focused on IS, with no identified clinical trials of nanomedicines for HS. This phenomenon may be attributable to the high prevalence of IS, the elevated production costs of nanomedicines, and the need for further validation of their biosafety. A patent has been developed for a liposome nanoparticle that responds to ROS and features a secondary targeting function for use in nanomedicines for stroke (CN116672325B). This invention also includes a method for preparing a peginterferon nanopreparation coated with a neutrophil membrane. The polymeric prodrug nanocarrier consists of a peginterferon polymer core surrounded by a neutrophil membrane shell, which is designed to respond to ROS in the tissue microenvironment of a stroke. This facilitates targeted delivery of the peginterferon to mitigate nervous system damage caused by local inflammation in IS; however, its targeting efficiency is currently inadequate.

**Additional Table 6:** Clinical trials of nanomedicine in stroke

Study title	NCT number	Status	Condition	Intervention	Sponsor	Study type
The effect of G D-iExo-003 in acute ischemic stroke	NCT06138210	Recruiting	Acute ischemic stroke	Drug: exosomes derived from human induced pluripotent stem cell for injection	Xuanwu Hospital	Interventional
Allogenic mesenchymal stem cell derived exosome in patients with acute ischemic stroke	NCT03384433	Unknown status	Cerebrovascular disorders	Biological: exosome	Isfahan University of Medical Sciences	Interventional
Australasian nanoparticle-mediated magnetically enhanced diffusion for ischemic stroke	NCT06495671	Not yet recruiting	Ischemic stroke	Device: Magnetically enhanced diffusion	University of Melbourne	Interventional

Additionally, a polypeptide for the treatment of IS (CN119039395A) has been developed, featuring the amino acid sequence YILREDGLGERTCLD. Another innovation is a co-assembled nanodrug for IS treatment (CN117398390A), which combines oleanolic acid and triptolide, with nanoparticle stabilization achieved using polyvinyl alcohol. This formulation reduces the hepatotoxicity and nephrotoxicity associated with triptolide while enhancing cerebral targeting due to its small particle size. It has demonstrated a protective effect against IS in animal models.

### The challenges facing the clinical application of nanomedicines

Nanomedicine has made substantial strides in stroke treatment; however, its practical implementation faces numerous challenges, primarily due to limitations in animal models. The existing preclinical investigations rely on animal models, such as mice and rats, which differ from human stroke in terms of pathophysiological pathways. For example, rodents have a lower ratio of white matter in the brain, limiting the simulations of white matter injury seen in humans following a stroke. Additionally, preclinical studies predominantly use young and healthy animals, whereas stroke patients often present with comorbidities such as hypertension and diabetes, which can influence responses to medication. Moreover, preclinical studies typically administer pharmaceuticals shortly after a stroke. In contrast, clinical treatment is often delayed for 6 h or more, resulting in missed opportunities for intervention during the ideal window. We propose that a composite model—integrating animals with atherosclerosis or diabetes—could more accurately reflect clinical conditions, while promoting multi-center standardized models to enhance data reproducibility. Another challenge lies in the design of clinical trials. The endpoints of preclinical investigations and clinical trials often show a disconnect. Preclinical stroke research primarily focuses on reducing short-term infarct or hemorrhage volume, while clinical trials are required to assess long-term functional recovery. Additionally, participants in clinical trials exhibit considerable heterogeneity, and the etiologies of strokes vary (including atherosclerosis and cardiogenic embolism), complicating the applicability of a single nanomedicine across all subtypes. In this context, clinical trials may benefit from employing biomarkers to identify target groups, mitigate the effects of heterogeneity, and simultaneously implement multi-target combination therapies to enhance efficacy.

The existing understanding of the influence of physicochemical properties of nanomedicines on their *in vivo* behavior remains inadequate. The process of optimizing the concentration and distribution of nanomedicines in target cells or tissues is complex. Preclinical models often fail to fully replicate the human physiological environment, limiting the reproducibility of clinical trial outcomes. No viable solutions to the challenges posed by nanoparticle toxicity and biocompatibility are available at present. We propose that the framework presented by Joyce, termed “DELIVER,” offers a suitable solution (Joyce et al., 2024). The researchers outlined seven principles: defined target product characteristics, critical attributes, optimization of lead medication candidates, proprietary intellectual property, validation of efficacy and safety, economic scalability, and regulatory and clinical pathways. The implementation of these principles, along with collaboration among all stakeholders, is essential to overcome the substantial challenges hindering the effective translation of nanomedicines. This includes issues related to nanomedicine characterization, preclinical evaluation, standardized testing, scalability and manufacturing economics, and regulation. We firmly believe that nanomedicines can achieve sustained progress in clinical translation.

## Safety

We examined the topic of surface modification of nanomedicines and assessed the current state of their therapeutic applications. The primary obstacle hindering the widespread clinical use of nanomedicines is their safety. This concern can be categorized into several sub-issues.

The first issue is cytotoxicity. Many nanomaterials, particularly carbon-based nanomaterials, can generate ROS upon entering cells. Excessive ROS can disrupt the redox balance within cells, and the resultant oxidative stress may cause cellular damage or death. The second concern pertains to DNA damage. Some nanomaterials can interact with DNA either directly or indirectly, resulting in DNA strand breaks or base damage and thereby compromising cellular function and genetic stability. For instance, carbon nanotubes, with their high aspect ratio and sharp edges, can directly interact with DNA and cause strand breakage. Fullerenes, with their unique spherical structure, can form complexes with DNA, altering its shape and affecting replication and transcription processes. Additionally, silver nanoparticles can release silver ions in the body, and an excess of these ions may be cytotoxic, potentially affecting DNA integrity. Lastly, lysosomes and mitochondria may show dysfunction. Nanomaterials can be internalized by lysosomes within cells, leading to lysosomal membrane rupture and the release of hydrolases, which may disrupt the intracellular environment.

The influence of the physical and chemical properties of nanomedicines presents another challenge. The surface chemistry of nanoparticles, including their surface charge and functional groups, affects their interactions with biomolecules. Additionally, as previously noted, the size and shape of nanomaterials significantly influence their biocompatibility. We argue that a contributing factor to these challenges is the lack of long-term toxicity research on nanomedicines. The chronic toxicity of many nanomaterials remains insufficiently studied, and longitudinal experimental evidence is lacking. As a result, the chronic toxic effects and carcinogenic risks associated with these materials are poorly understood, making a comprehensive assessment of their long-term consequences on the body unfeasible. To address safety concerns, comprehensive research covering three key areas is essential: first, surface functionalization technology; second, biocompatibility evaluation; and third, standardization of the manufacturing process. Thus, establishment of rigorous manufacturing practices and quality control requirements while simultaneously achieving large-scale production of nanomaterials is essential. Ultimately, long-term research on nanomedicines must be prioritized. This will necessitate comprehensive toxicity and safety studies to evaluate the prolonged effects of nanomaterials in the body, including chronic toxicity, effects on the immune system, and reproductive and developmental toxicity. Additionally, clinical studies must monitor patients’ health over an extended period to assess the long-term safety and efficacy of nanomaterials, thereby establishing a scientific foundation for their clinical applications. We believe that these strategies will enhance the prospects of nanomedicines in stroke treatment.

## Limitations

This review article had certain limitations that require consideration. First, references regarding clinical studies of nanomedicines for stroke, FDA new drug approvals, and NIH funding are limited. The existing research predominantly focuses on animal experimentation. Second, numerous treatment avenues for stroke have not been detailed in this article.

## Summary and Outlook

This article thoroughly examines the current state and prospects for the use of nanomedicines during the rehabilitation phase of stroke. The post-stroke rehabilitation process is complex and prolonged. Moreover, existing clinical interventions face numerous challenges during the subacute and chronic phases, with the inadequate restoration of the BBB restricting the cerebral transport of pharmaceuticals. Advancements in nanomedicine have introduced novel approaches for stroke treatment. Nanoscale-level modification of conventional pharmaceuticals or adjustment of the delivery routes, such as through intravascular injection or intranasal administration, can significantly enhance a drug’s ability to penetrate the BBB, target specific sites, and improve bioavailability. Despite the substantial promise of nanomedicines in stroke treatment, several hurdles and issues need to be addressed in future research. The first concern is the ability of nanomedicines to effectively penetrate the BBB.

The design of nanomedicines requires further optimization to enhance its ability to traverse the BBB and ensure optimal drug delivery to the lesion site. Stability and biocompatibility are essential for their therapeutic application. Future studies should focus on improving the stability of nanomedicines, mitigating immune responses and potential toxicity, developing nanomedicines with active targeting capabilities for precise delivery to the lesion site, and exploring controlled-release mechanisms to reduce adverse reactions and enhance efficacy. Comprehensive safety evaluations of nanomedicines before clinical application, including long-term *in vivo* distribution, metabolism, and potential toxicity studies, are necessary to ensure safety for clinical translation. Additionally, interdisciplinary collaboration should be actively pursued. The advancement of nanomedicines requires collaborative efforts among experts in materials science, pharmacy, biology, and clinical medicine to facilitate the transition from laboratory research to clinical application.

In summary, nanomedicines hold substantial potential for use during the post-stroke rehabilitation phase. However, numerous scientific and technical challenges must be addressed before the application of nanomedicines in clinical practice. We believe that continued research and innovation will enhance the role of nanomedicines in future stroke treatment, ultimately leading to improved rehabilitation outcomes and a better quality of life for patients.

## Additional files:

***Additional Table 1:*** *Application of related nanomedicines in stroke management.*

***Additional Table 2:*** *Nanozyme-related applications in ischemic stroke.*

***Additional Table 3:*** *Advantages and disadvantages of different administration methods and their application.*

***Additional Table 4:*** *Relevant surface modifiers and their functions in stroke.*

***Additional Table 5:*** *Animal models of stroke.*

***Additional Table 6:*** *Clinical trials of nanomedicine in stroke.*

## Data Availability

*All relevant data are within the paper and its Additional files*.
